# Stakeholder perspectives on the scalability of a psychological intervention for alcohol misuse and psychological distress in wartime: A qualitative study in Ukraine

**DOI:** 10.1371/journal.pmen.0000639

**Published:** 2026-07-07

**Authors:** Khatia Antia, Sergiy Bogdanov, Olha Biklian, Vita Kachai, Catharina van der Boor, Bayard Roberts, Kostyantyn Dumchev, Daniela Fuhr

**Affiliations:** 1 Department of Prevention and Evaluation, Leibniz Institute for Prevention Research and Epidemiology - BIPS, Bremen, Germany; 2 Centre for Mental Health, National University of “Kyiv-Mohyla Academy” (NaUKMA), Kyiv, Ukraine; 3 Department of Health Services Research and Policy, London School of Hygiene and Tropical Medicine (LSHTM), London, United Kingdom; 4 Health Sciences, University of Bremen, Bremen, Germany; Australian National University, AUSTRALIA

## Abstract

The war in Ukraine has intensified mental health vulnerabilities, including alcohol misuse among conflict-affected men. The CHANGE intervention, a transdiagnostic mental health programme building on WHO’s Problem Management Plus (PM+), addresses alcohol misuse and common mental disorders among conflict-affected populations. This study explores stakeholder perspectives on the scalability of CHANGE under active wartime conditions in Ukraine. Here, scalability refers to the potential for future scale-up rather than retrospective evaluation of actual scale-up, feasibility or effectiveness. Guided by the Consolidated Framework for Implementation Research (CFIR), we conducted online interviews with 20 stakeholders: 13 implementers, 2 adopters, and 5 maintainers. Perceived barriers in the *outer setting* included limited primary care referrals, lack of policy integration, societal stigma, normalization of alcohol use, competition among service providers, intersectoral trust gaps regarding NGOs and funding instability. *Inner setting* barriers included psychological distress among implementers and payment instability. Perceived facilitators across CFIR domains included established multisectoral partnerships, a supportive organizational environment, team professionalism, and strong motivation for implementation. The war introduced additional barriers, including service disruptions, insecurity and economic hardship. At the same time, the online adaptation of the intervention, and increased community engagement around mental health needs emerged as key facilitators. Suggested implementation strategies focused on strengthening stakeholder relationships, ensuring continues training and supervision, and engaging service users through awareness campaigns. Overall, findings from CHANGE provide contextually grounded insights for scaling psychological interventions in humanitarian and conflict-affected settings globally.

## Introduction

Psychoactive substance use poses major global health challenges, with alcohol use disorders (AUDs) among the leading causes of premature death and disability, accounting for over 3 million deaths annually [1]. The harmful effects of alcohol disproportionally affect people living in vulnerable situations, particularly those with limited access to health and social care [1–3], and are further aggravated in humanitarian crises. During war and prolonged adversity, individuals often resort to harmful coping mechanisms such as excessive alcohol use, contributing to increased psychosocial distress and health risks [4–7].

In Ukraine, these challenges have intensified due to ongoing war, widespread displacement, and disruption of health and social infrastructure [8–11]. Since the escalation of war in February 2022, more than 12 million people have required humanitarian assistance [12]. Elevated psychological distress, and alcohol misuse, especially among men exposed to combat trauma, and economic hardship, have been widely documented [3,13]. National survey data confirm substantial gender differences, in alcohol consumption, with men consuming an average of 14.7 grams of pure alcohol daily (~1.5 Standard Units), compared to 3.1 grams among women (~0.3 Standard units) [14]. A recent qualitative study further suggests that alcohol use functions as a form of self-medication for trauma and hopelessness [3].

Ukraine’s mental health system, historically reliant on inpatient psychiatric care with limited integration at community and primary health care (PHC) levels [15–17], is undergoing a major reform accelerated by war realities [18]. Recent national initiatives, such as the “How are you?” mental health program [19], the Mental Health and Psychosocial Support (MHPSS) Technical Working Group [18,20], the Coordination Centre for Mental Health [21], and the Action Plan for 2024–2026 for mental Health development [22], reflect growing governmental and intersectoral commitment to mental health reform. Nevertheless, community-based services remain underdeveloped, with psychiatric hospitals still forming the core of care [16,23]. The Lancet Psychiatry Commission has therefore called for rapid, scalable mental health solutions delivered by trained non-specialists within stepped-care models, accompanied by sustained investment in training, research and policy reform [17].

The WHO’s Mental Health Gap Action Programme (mhGAP) promotes brief psychological interventions as frontline responses in humanitarian and in low-resource settings [24,25]. Research supports the effectiveness of short-term, community-based interventions delivered by trained lay health workers for displaced and conflict-affected populations [26,27]. In such contexts, where formal health systems are disrupted, the non-governmental sector plays an essential role in implementing mental health interventions [28]. Successful collaborations, such as the STRENGTH project [29], illustrate how partnerships among research institutions, international agencies and NGOs can adopt and deliver psychosocial interventions across diverse humanitarian contexts.

While these interventions show promise, significant challenges persist when it comes to their scaling up. *Scaling up* is defined as the “deliberate efforts to increase the impact of successfully tested pilot, demonstration or experimental projects to benefit more people and to foster policy and programme development on a lasting basis” [30]. Although implementation and scaling up share common determinants, they are analytically distinct: Unlike routine implementation or feasibility testing, scaling up requires system-level embedding, including national policy integration, public financing, primary care referral pathways to achieve population coverage [31]. Assessing scalability alongside effectiveness trials can reveal intervention’s suitability for broader integration [31]. However, evidence on how mental health programmes can be effectively scaled in contexts of forced displacement and ongoing armed conflict remains critically limited [32]. Existing studies [32–34] have largely focused on post-conflict or refugee settings, leaving a substantial gap in our understanding of scalability under conditions of active war, where health systems are simultaneously disrupted reformed and overwhelmed.

This evidence gap is particularly consequential. In active conflict settings, the barriers to scale-up are likely to extend beyond those documented in stable or post-crisis contexts [33], encompassing not only structural challenges, but also dynamic war-specific factors that have received little systematic attention in implementation research [32]. Qualitative approaches are particularly well-suited to surface such contextually specific factors, and inform the design of sustainable scale-up strategies.

Implementation science frameworks offer a promising, yet underutilized approach to optimize intervention design, implementation and scale-up in humanitarian settings, where resource constrains and rapid response needs demand systematic adaptation of health strategies [35]. Frameworks such as the Consolidated Framework for Implementation Research (CFIR) provide a structure for analysing barriers and facilitators influencing intervention uptake [36,37]. CFIR organizes determinants into five domains: intervention characteristics, outer setting, inner setting, characteristics of individuals, and implementation process, illustrating how factors such as organizational readiness, policy pressures, and stakeholder engagement interact in real-world settings [36,37]. Complementing CFIR, the Expert Recommendations for Implementing Change (ERIC) taxonomy offers a structured compilation of 73 discrete strategies, organized into nine thematic clusters [38–40].

Both, CFIR and ERIC has proven valuable in resource constrained and conflict-affected contexts [41–46]. Importantly, the two frameworks serve distinct but complementary analytical functions: CFIR identifies and organizes the contextual determinants that influence whether and how an intervention can be implemented and scaled, while ERIC provides strategies for addressing those determinants in practice. This structured approach is particularly valuable in war contexts, where implementation determinants are highly dynamic and strategies must be both evidence-based and contextually responsive. CFIR has informed mental health implementation studies examining adaptation, implementation and sustainability of interventions [44,45], while ERIC has enabled tailoring of discrete strategies, such as stakeholder engagement, capacity building and workflow adaptation [35,46,47].

This study is part of a broader CHANGE project “Alcohol use in humanitarian settings: A programme of work to address alcohol misuse and associated adversities among conflict-affected populations in Uganda and Ukraine” [48–50].

Building on the broader scope of the CHANGE project and its existing evidence base, this study qualitatively explores stakeholder perceptions of factors that may influence the scalability of the CHANGE intervention under wartime conditions in Ukraine. Consistent with established definitions, [51,52], *scalability* here refers to potential scale up of CHANGE, rather than retrospective evaluation of actual scale-up, feasibility or effectiveness. The study specifically aims to: (i) identify perceived barriers and facilitators influencing the implementation of CHANGE within the non-governmental sector amid ongoing war; and (ii) explore implementation strategies and contextual factors that stakeholders consider critical for supporting future scale-up. Findings from this study will directly inform a subsequent Theory of Change workshop, in which stakeholders will collectively develop a structured scale-up pathway for CHANGE based on the evidence generated here. In this study, we use CFIR to structure our exploration of contextual determinants, and draw on the ERIC compilation of implementation strategies to interpret stakeholders’ suggested actions for improving implementation and potential scale-up. This study is expected to generate contextually grounded insights that can guide efforts toward sustainable mental service expansion in humanitarian and war-affected settings.

## Materials and methods

### Ethics statement

This study adheres to key ethical principles for the conduct of research following international guidance (e.g., Declaration of Helsinki), and specific guidance for humanitarian settings (e.g., for R2HC by Elhra), the London School of Hygiene & Tropical Medicine (LSHTM)’s ‘Good Research Practice Policy’, LSHTM’s data protection principles and its ‘Data Protection Policy’. This study also follows the recommendations for conducting ethical mental health and psychosocial research in emergency settings as suggested by the Inter-Agency Standing Committee (IASC) [53] Reference Group for MHPSS in Emergency Settings.

The study received ethical approval from the ethics committees of the London School of Hygiene & Tropical Medicine LSHTM- Approval Number: 31251, and the ethics committee of National University of Kyiv-Mohyla Academy NaUKMA - Approval Number: FWA00030125.

Written informed consent (digitally signed) was obtained from all participants prior to scheduling the interviews.

### The CHANGE intervention

The CHANGE intervention is designed to addresses common mental health comorbidities such as alcohol misuse, depression, anxiety, and post-traumatic stress disorder among conflict-affected populations [50]. In Ukraine, the intervention is delivered remotely to war-affected men across all government-controlled territories through six weekly 90-minute sessions conducted via Zoom. Using motivational interviewing techniques, facilitators guide participants through three structured phases: engagement and psychoeducation, behaviour change and coping strategies, and relapse prevention. Sessions are delivered by trained facilitators [54].

To support implementer well-being and performance during wartime conditions, weekly 90–120-minute remote group supervision sessions were held for facilitators, led by a trained supervisor. These sessions addressed case discussions, emotional support from peers/supervisors, and safety protocols, with individual supervision available as needed. In addition to client safety sessions, data collectors received weekly team calls for work reflection and peer support. Supervisors held weekly clinical exchange calls with a dedicated group chat for urgent issues and workload flexibility.

The intervention is implemented by the National University of Kyiv-Mohyla Academy (NaUKMA) Mental Health Center and NGO “WordsHelp” Ukraine within pilot and definitive trials.

### Study design

A qualitative study design was employed to explore the scalability of the CHANGE intervention.

We conducted individual key informant interviews with participants based on their roles in implementing, adopting, and maintaining the CHANGE intervention. In this study, *implementers* refer to individuals directly involved in putting the CHANGE intervention into practice, responsible for executing specific activities related to its implementation (e.g., supervisors, facilitators). *Adopters* refer to individuals or institutions that support the integration of the CHANGE intervention into existing practices or systems (e.g., the NaUKMA Mental Health Center, and NGO WordsHelp). *Maintainers* are those who may be involved in ensuring the long-term sustainability and continued delivery of the CHANGE intervention by gradually embedding it into routine practice in the future (e.g., members of government agencies, NGOs, and international organizations).

### Sample

We purposively recruited stakeholders critical to the CHANGE intervention’s implementation and potential scale-up. Prior to the study, we compiled an initial list of potential implementers, adopters and maintainers. The list was finalised in discussion with the project team. Sampling targeted specific, often singular roles essential to program delivery. We therefore prioritized maximum variation across roles and stakeholder perspectives rather than thematic saturation, following established approaches for key informant studies in implementation science [55–57].

Implementers were chosen based on their roles and hands-on experience with the implementation and delivery of the CHANGE intervention. To ensure a broad and diverse range of perspectives, individuals at all levels of involvement were invited, from data collectors to the research manager.

For adopters, we invited representatives from both the NaUKMA Mental Health Centre and NGO WordsHelp. Maintainers included representatives from organizations actively engaged in providing psychosocial support to individuals facing psychological distress and psychoactive substance use (e.g., Public Health Centre of the Ministry of Health, Ukrainian Red Cross). The list also encompassed organizations with the capacity to offer funding or other essential resources, such as disseminating information about CHANGE, and supporting referral pathways (e.g., United Nations Office on Drugs and Crime, Kyiv City Center for Social Services).

Invitations to participate in the study were sent via email, which included digital copies of the study information sheet and informed consent forms, along with the contact details of the research manager (V.K). Non-responders received a reminder email, and clarifications were provided by phone when requested.

### Topic guides

The interviews were guided by topic guides developed based on relevant domains of CFIR [37,58]. We designed three distinct topic guides to address the specific perspectives of: (i) implementers, (ii) adopters, and (iii) maintainers of the CHANGE intervention. The guides covered key aspects of the CFIR framework: (i) key conceptual factors that influence adoption, implementation and maintenance of the CHANGE intervention; and (ii) potential barriers and facilitators that magnify when scaling up CHANGE.

Additionally, we explored participants’ perspectives on specific implementation strategies that could help address the barriers identified by participants during the interviews. As a structured framework for probing implementation strategies, we followed Powell and colleagues’ ERIC taxonomy [38–40].

All questions and probes were tailored to the context of Ukraine, considering the social, political, and economic landscape. An overarching question was also included to explore the impact of the ongoing war, and its potential influence on CHANGE implementation. Topic guides were piloted prior of being used in the study. An example of a topic guide (for implementers) is available in S1 Appendix.

### Data collection

This study was conducted remotely across Ukraine, considering the heightened security concerns due to ongoing war. Data collection took place between November 1 and December 18, 2024. We conducted a total of 20 interviews; the sample consisted of (i) programme implementers (N = 13): data collectors, facilitators, supervisors, a project manager, and a recruitment officer. (ii) programme adopters (N = 2): representatives of NaUKMA and WordsHelp and (iii) maintainers (N = 5): national health officials including representatives from the Ministry of Health and Kyiv City Centre for Social Services.

Interviews were conducted online via Zoom, in either English or Ukrainian, based on participant preferences. As data collection occurred during active war in Ukraine, despite reliable internet and electricity access for most participants and interviewers, occasional power outages required rescheduling some interviews. All interviews were audio recorded (Zoom recordings), but the recordings were deleted after transcription was completed. Of the 20 transcripts, 5 were already in English, and the remaining 15 Ukrainian transcripts were translated into English by the same certified translator service. Translation accuracy and meaning equivalence were verified by the third author (O.B.), a native Ukrainian speaker with advanced English proficiency who conducted all Ukrainian-language interviews. All transcripts were fully anonymized. Participants were asked to give explicit consent for the use of anonymized quotes in publications and other forms of knowledge dissemination.

### Data analysis

We analysed qualitative data using a thematic analysis approach based on the methodology of Braun and Clarke [59]. The first (K.A.) and last (D.F.) authors developed an initial deductive coding framework from the CFIR [58], but inductive coding was also employed to capture themes that emerged directly from the data. The CFIR-guided coding framework and summary of codes are provided in Supplementary Materials S2 Appendix and S3 Appendix. Additionally, we categorized the implementation strategies suggested by participants according to Powell and colleagues’ [38,39] ERIC discrete implementation strategies. Using MAXQDA 2020 [60] qualitative data analysis software, K.A coded and organized the data into key categories. Initial codes were discussed with the third author (O.B.), ensuring linguistic and contextual validity. K.A. then presented preliminary findings to the last author (D.F.) in weekly meetings for reflexivity and refinement. Final themes and sub-themes were decided collectively through by-weekly reflexive discussion meetings with all co-authors.

### Reflexivity statement

The first author (K.A.), a Georgian researcher based in Germany who joined CHANGE in 2024, conducted 5 English interviews with limited prior contact (one adopter, one implementer). The third author (O.B.), a Ukrainian researcher at NaUKMA Mental Health Centre, conducted 15 Ukrainian interviews while maintaining neutrality through a 2-year international fellowship absence from the Centre; she had no prior contact with 8/15 respondents, with remaining contacts ranging from general familiarity (n = 4) to prior team collaboration (n = 3). The fourth author (V. K.), NaUKMA researcher, and self-identified insider, facilitated recruitment without coercion. The last author (D.F.), senior implementation researcher, provided oversight of analysis through weekly reflexive meetings without data collection involvement. We acknowledged potential influences from programme familiarity and institutional affiliations, addressed through weekly reflexive meetings, and multi-author validation.

## Results

Most participants who were interviewed identified as female (N = 16) and were based in or resided in central Ukraine, including fourteen in Kyiv, one in Vinnitsa, and one in Poltava. Four participants lived in Dnipro, in eastern Ukraine. Interview durations ranged from 41 to 104 minutes. A detailed overview of participant characteristics is provided in S4 Appendix.

Study findings are organized according to the CFIR domains: outer setting, inner setting and individuals. The analysis also highlights sub-themes reflecting both, CFIR framework constructs and emergent data patterns, such as barriers specifically related to the ongoing war and their potential impacts. The section concludes with proposed implementation strategies offered by participants, aimed at enhancing the future scale-up of the CHANGE intervention.

### Implementation barriers and facilitators

Here, we outline implementation barriers and facilitators as perceived by the stakeholders. A detailed summary is provided in Table 1.

**Table 1:** Summary of perceived implementation barriers and facilitators in the CHANGE implementation, organized by CFIR domains.

CFIR Domain	Construct name	Barriers to Implementation	Facilitators of Implementation
**I. Outer Setting**	**B. Local Attitudes** **D. Partnerships & Connections**	• Lack of referrals from primary healthcare, e.g., family doctors • Limited public awareness of health risks associated with AUD • Limited public awareness of psychological interventions, including the CHANGE programme • Limited support from some potential partners (prioritize their own agendas or view CHANGE solely as a research initiative, intersectoral trust gaps regarding NGOs) • Insufficient awareness of the CHANGE content • Competition among service providers • War-related barriers (see detailed report in Table 2)	• Local community organizations regarded as reliable partners • Availability of CHANGE to provide referrals • NGO support to raise public awareness (e.g., disseminating information about CHANGE) • CHANGE’s capacity to shift from face-to-face to fully online delivery due to the war • Increased relevance of CHANGE during the war • Nationwide recognition and respect for the implementing institution (NaUKMA)
	**E. Policies & Laws**	• CHANGE not being included in clinical standards of care (developed by the Ministry of Health) due to its not yet fully demonstrated effectiveness and limited evidence base.	• Established referral procedures with existing partners • Favourable political environment - government instructions to address AUD
	**F. Financing**	• Limited opportunities to attract grants • Lack of long-term financing • Economic instability due to war	• Targeted funding, with political will from some governmental agencies to increase programme funding.
	**G. External pressure**	• Societal stigma about mental health issues and AUD • Normalization of alcohol use and AUD	• Public awareness campaigns (e.g., social media campaigns) • Advocacy from veteran and MHPSS group-member organizations
**II. Inner Setting**	**A. Structural** **B. Characteristics** **D. Culture** **J. Available Resources**	• Worsened payment system for implementers (unstable payments and salary decreases) • Psychological distress among the CHANGE implementation team (due to the war)	• Supportive work environment • Clear organizational structure • Regular supervision • Flexible work schedule
**III. Individuals (providers)**	**Roles subdomain** **D. Implementation Facilitators** **F. Implementation team members** **Characteristics subdomain** **C. Opportunity** **D. Motivation**	• None Identified	• Strong team dynamics and shared mission within the team • Access to necessary resources • Continuous training and supervision • Team’s prior experience and professionalism • Opportunities to be involved in research • Respect for NaUKMA as the implementing institution • Motivation driven by the social relevance of the topic and helping those in need

#### Outer setting.

In the CFIR framework, the *outer setting* refers to the external context that influences an organization’s implementation efforts, encompassing economic, political, social, and environmental factors that can impact adoption, implementation, and sustainability of an intervention [37,58]. Within this domain, we examined key sub-constructs including local attitudes, partnerships and connections, policies and laws, financing and external pressure.

In addition to war-related challenges (reported separately; see Table 2), stakeholders identified several interconnected barriers constraining scalability of CHANGE.

**Table 2:** Implementation Barriers related to the war and their potential impact.

Barrier Category	Detailed Description	Potential Impact	Demonstrative Quote
**Disruption due to Mobilization/Migration**	Constant mobilization efforts and population displacement (internal and external) making it difficult for individuals to attend sessions.	Interrupted treatment	*If we take a full-scale war, this is exactly what influences, does not give a 100% guarantee that tomorrow the consultation will take place, for technical reasons, perhaps from the safety situation […] this person may simply not attend the session at some point, because they were mobilized, right? Well, the changes are constant. And even those clients who attend, they move constantly [change the place of residence]. (Implementer 8)*
**Safety Concerns**	Ongoing war prevents sessions from taking place, putting both clients and implementers at risk.	Missed appointments	*We cannot manage good circumstances for clients. Also, this power outages. And yeah, I mean, or if another attack happening like you can just stop and ensure person following some safe space, but we cannot even guarantee that person will reach that safe space, but I mean, yeah, there are things we can’t influence on our project level. (Implementer 2)*
**Low Recruitment Rates due to Fear**	Men avoid participating in interventions due to fear of being conscripted into military service.	Low Recruitment	*Men are hiding, some men who are not at the front and who have not been resigned because of illness or for some other reason - they are just hiding at home. They’re afraid to even share their data there, even though you keep saying that we don’t share this [...] we have stated that our information is not shared to any government agencies. This avoidance of mobilization is just a nightmare, and they are, well, to some extent, so intimidated that they are afraid to say their name. (Implementer 11)*
**Lack of Access and Resources**	Loss of electricity/internet access hinders online participation.	Exacerbated isolation, reduced treatment adherence.	*Well, I’m not even talking about the fact that we solve problems with electricity for counsellors, right? For example, we have Starlink. But on the other hand, a person does not have it, and they cannot be on the session. (Adopter 13)*
**Economic Hardship and Funding Instability Reduced Prioritization of Mental Health**	Resources diverted to war efforts creating uncertainty around long-term funding; War worsens economic inequalities.	Programme disruptions	*Lack of money is the one, because, yeah, sustainability means who pays, and Ukraine is right now, yeah, its very bad economic conditions, we rely on an external funder, and that is the question. Would it be a priority for external funders or not? And what exactly they want to support, would they? (Adopter 5)*
**Psychological distress among providers**	CHANGE implementation team may develop psychological distress due to war and may be unable to deliver the intervention.	Lack of human resources	*You yourself are in a situation where you need psychotherapy, intervention, or something else. Well, I mean, it’s really an obstacle. We are not robots, we are all under the same sky here in Ukraine, and every massive shelling is stressful for all of us. And it feels like you just start from scratch, always from scratch. And it’s very difficult, you organize space from scratch, you organize yourself from scratch, you put yourself in order. (Implementer 9)*
**Erosion of Trust**	Prolonged war erodes trust in institutions.	Increased scepticism towards interventions	*You see, well, war has a very hard effect on everything, tension in society is growing and despair is growing. And the longer this war lasts, the greater the exhaustion and despair. Well, more people aim for a life of one day. And, accordingly, in search of quick, easy pleasures. The longer this goes on in society, the less trust people will have in any institution. (Implementer 3)*
**Loss of Motivation and Hope**	Overwhelming challenges decrease help seeking.	Decreased engagement in treatment and early dropout.	*We have less clients because of some internet connection issues. Sometimes we have electricity issues, not because we not try to contact our clients different time or different day. It is because they lose their motivation if it is not too strong to beat these obstacles. (Implementer 1)*

**Limited primary care referrals, societal stigma and awareness gaps.** Limited primary care referrals were not described as standalone issue but as the results of interacting societal and system-level barriers. Across stakeholder groups, participants emphasized that societal stigma surrounding mental health, the normalization of alcohol use, and limited public awareness of AUD health risks and available psychological interventions collectively undermined help-seeking. These factors contributed to low problem recognition, with individuals often failing to identify problematic alcohol use at an early stage, as described by one implementer: *“Turning a blind eye, turning a blind eye to the problem” (Implementer 11).* Family doctors were seen as particularly reluctant due to time pressures, discomfort with stigmatized topics, and avoidance of emotional discussions, as one adopter explained: *“Stigma plays a big role that they not start speaking about this problem. […] it is also unpleasant topic to discuss with a patient”. (Adopter 5).* Participants emphasized the need for broader awareness-raising about programmes like CHANGE to overcome these entrenched attitudes.

**Policy exclusion, NGO legitimacy concerns, competition among service providers and CHANGE’s positioning as a research initiative**. Although state organizations were generally viewed as supportive, the absence of formal policy integration emerged as key barrier of scalability. CHANGE is perceived as research initiative, with its effectiveness yet to be fully demonstrated; as such, it has not been prioritized or included in governmental standards of care. As one governmental stakeholder explained:

*We initiate the writing of standards; we initiate the introduction of changes. Therefore, as for political support, it can be here, if there is evidence of the effectiveness that we would like to receive, and some kind of plan to discuss the implementation of opportunities, for example, scaling up this intervention. (Maintainer 15)*

Implementers echoed this, citing exclusion from standards as a key reason for limited family doctor referrals despite active outreach efforts. Compounding this, some governmental maintainers expressed scepticism toward NGOs as long-term implementation partners, perceiving them grant-dependent and raising concerns about staff qualifications and licensing in the non-governmental sector. *“And today, as a state, we would still move more towards healthcare institutions, and gradually establish, but not immediately scale it to NGOs.” (maintainer 15).*

At the same time, limited understanding of the CHANGE among potential partners, particularly confusion between its research and service delivery functions, created uncertainty about programme’s role. This occurred alongside a broader proliferation of NGOs providing psychological services since the full-scale invasion, increasing competition, service overlap, and client confusion: “*In time of full-scale invasion, we have more NGOs opened and they provide more services. Now almost every NGO provides psychological services” (Implementer 11).*

**Funding instability threatening sustainability.** All stakeholder groups identified short-term funding cycles, war-related economic pressures, and insufficient mental health care budgets as critical barriers to long-term planning and intervention delivery. As one maintainer noted:

*If you look at how psychiatric care is funded, it is a psychiatric package of services, […] It is insufficient, it is very small. And outpatient care, it is also insufficient and very small And, accordingly, healthcare facilities may not be very interested in delivering such interventions. (Maintainer 15)*

Stable financing was viewed as curtail for transforming any intervention from pilot to population level-impact.

**Facilitators identified by stakeholders**: Despite barriers described above, implementers and adopters highlighted reliable partnerships with local community organizations as key facilitator, providing expertise, referrals, and ties to national entities. NGO-led public awareness campaigns through platforms such as Facebook and Telegram alongside advocacy by MHPSS groups and veteran organizations were seen as fostering greater community acceptance of CHANGE: *“It is great when the NGO sector takes on this leadership role, when it takes on the task of advocating this topic and moving it forward, influencing local authorities to create appropriate social services. (Maintainer 19)* Some governmental stakeholders also noted potential for increased funding, contingent on evidence of demonstrated effectiveness and political will.

#### Inner setting.

The *Inner Setting* refers to features within the implementing organization that influence implementation outcomes, including organizational characteristics, culture, and available resources [37,58]. Within this domain, we examined adopters’ and implementers’ perceptions of task organization, responsibilities, shared values, and resource availability in relation to CHANGE delivery.

All adopters were affiliated with the NaUKMA Mental Health Center and NGO WordsHelp, and all implementers were employed by these organizations as part-time project staff within the research trial. As such, participants were not able to comment on organization-specific barriers in routine service settings, as many were employed by institutions that had not yet implemented CHANGE. This limitation is relevant for scalability assessments, as inner setting conditions during a research trial may differ substantially from those in future implementing organizations.

**Workforce strain as a scalability risk**. The most consistently raised inner setting concern was the psychological impact of the ongoing war on the implementation team. While implementers considered themselves currently capable of delivering CHANGE effectively, participants expressed concerns that escalating war stressors, including shelling and chronic uncertainty, could worsen their mental well-being and progressively erode their capacity to continue service provision. Payment instability compounded this concern, with some implementers noting salary decreases during the implementation period, raising concerns about staff retention as the programme extends. Together, these workforce pressures were perceived as posing a tangible risk to sustained programme delivery, particularly if implementation moved beyond the current team.

**Organizational facilitators supporting implementation quality.** Participants consistently described organisational conditions that actively supported implementation. A clear role structure, regular supervision, flexible scheduling, and strong team cohesion were described as enabling consistent intervention delivery under wartime conditions. As one implementer explained: *“for the research team, we established roles like field team leads, regular supervision, and emotional support, which is important, even for data collectors working with clients.” (Implementer 2)*. Management provision of necessary equipment, including computers, tablets, internet connectivity, and office spaces with backup power, ensured session connectivity, even during electricity disruptions. As one of the implementers expressed: *“I always knew that I have an office in my city. I can go to where there is a power bank, and I have Internet connection. And I am ready to have my sessions with clients anytime I need”. (Implementer 3)*

Overall, the findings indicated that current organizational environment is actively enabling intervention delivery; however, sustained strategic and operational support from NaUKMA and WordsHelp was identified by participants as essential for ensuring the programme’s sustainability and scalability.

#### Individuals domain.

The *Individuals* domain refers to the characteristics of persons involved in implementing or experiencing the intervention, including their knowledge, beliefs, self-efficacy, motivation, and other personal attributes that influence their engagement and performance [37,58]. Within this domain, we examined the extent to which implementation team members had the opportunity and motivation to effectively fulfil their roles.

No barrier was identified in this domain. As described above, all participants were embedded in a well-structured research trial with regular supervision and institutional support. In addition, interviews were conducted by members of the research familiar to some participants, which may have shaped how individual-level barriers were reported.

**Team professionalism and contextual expertise.** Implementers consistently framed team professionalism and contextual expertise as key facilitators of implementation. Prior experience, familiarity with the Ukrainian context, and knowledge of relevant organizations and referral pathways were seen as reinforcing both confidence and credibility in delivery. As one implementer described:

*Professional team, for sure. I see (anonymized names) that they are really professionals, I mean their expertise is wider than addictions, for instance. They know the context of Ukraine really well, they know people, they know lots of organizations and individuals with whom they can refer to them. (Implementer 3)*

**Motivation and sustained engagement**. Implementers highlighted the novelty and social relevance of the intervention, as well as a sense of professional recognition associated with NaUKMA, as key drivers of initial engagement. As one implementer explained: *“It is interesting experience to be part of something to help, to create something very useful for people. […] and it is big honour to be a part of the team who try to make a difference. (Implementer 2).* These motivations were closely tied to the perceived societal value and timeliness of the intervention in the Ukrainian context. Sustained engagement, however, appeared to depend more on ongoing organisational support.

### Implementation barriers related to the war and their potential impact

The ongoing war introduced a distinct layer of implementation challenges that cut across all CFIR domains. Unlike the structural and contextual barriers discussed above, war-related barriers were characterised by their unpredictability and the limited extent to which they could be addressed at programme level. Key barriers included mobilization and displacement disrupting client engagement, safety concerns causing missed appointments, fear of conscription reducing male recruitment, electricity and internet outages limiting online access, and economic hardship threatening programme sustainability. Despite these challenges, participants noted growing community recognition of mental health and AUD support needs as emerging opportunity, and identified full shift to online delivery as critical adaptive response enabling continuous access. Detailed descriptions, potential impacts, and illustrative quotes for each barrier are provided in Table 2.

### Implementation strategies

Powell and colleagues define *implementation strategies* “a systematic intervention process to adopt and integrate evidence-based health innovations into usual care” [38]. Participants identified strategies primarily within 3 of the 9 ERIC implementation strategy clusters conceptualised by Powell et al [39,40]: *Develop Stakeholder Interrelationships, Train and Educate Stakeholders,* and *Engage Consumers*. Below is a brief overview of these strategies, with more detailed descriptions available in S5 Appendix.

Developing stakeholder relationships emerged as a central strategy across participant groups. Partnerships with community organizations, governmental actors, and international entities were repeatedly described as key to facilitating implementation and enabling future scale-up. Strategies included engaging regional agencies, employing community consultants, mapping organizations with aligned goals, and maintaining collaboration with local actors to facilitate participant recruitment and communicate CHANGE’s evidence-based effectiveness (currently under evaluation). As one participant suggested:

*So, you may go across the NGOs, who already have social workers, who have the clients, have the network across Ukraine, or in the region, for example, and considering this, I would address the state-run social services on municipal level as well. (Maintainer 4)*

Within the training and education cluster, key approaches included providing ongoing training and consultation, and utilizing collaborative training methods, such as train-the-trainer models and partnerships with educational institutions. These efforts were viewed as essential to embed CHANGE within existing educational frameworks and ensure sustained supervision.

Strategies related to engaging consumers were closely linked to addressing stigma, limited programme awareness, and referral barriers. Stakeholders recommended mass media campaigns, outreach through frontline professionals and family doctors, clearer service-focused messaging, high-level advocacy, and demonstrating international best practices. Community networks and faith-based organizations were also identified as important channels for reaching marginalized populations.

Cross-cutting strategies related to financial sustainability and system integration were also identified. Maintainers suggested approaches such as municipal co-funding, attracting donor support, and clarifying payment sources for facilitator training and supervision. Participants also emphasized ongoing monitoring and evaluation, learning from comparable programmes, and fostering collaborative, rather than competitive, relationships among service providers as relevant to implementation, sustainability and resilience.

## Discussion

This qualitative study expands implementation research from other conflict settings to Ukraine’s active wartime context, examining perceived factors influencing the scalability of a psychological intervention with a focus on barriers, facilitators and suggested implementation strategies for future scale up. Stakeholders identified key barriers of implementing CHANGE in Ukraine included limited support, lack of primary care referrals, low public awareness of alcohol-related health risks and psychological interventions, societal stigma, normalization of alcohol use, financial constraints, competition among service providers, and war-related challenges. Key facilitators included established partnerships with local and national organisations, supportive work environment, and team professionalism. The ongoing war further complicated implementation by raising concerns over data confidentiality, mobilization fears, and economic hardships. Conversely, adaptive strategies, such as transitioning to online platforms were perceived as effective in maintaining progress. Stakeholders recommended strategies to support scale-up of CHANGE including developing stakeholder interrelationships, training and supervision, targeted awareness campaigns, and utilizing existing networks for outreach and dissemination.

Despite the urgent need for interventions addressing AUD and psychological distress in humanitarian settings [50], evidence on their scale-up and factors associated with it is still limited. Existing literature emphasizes the importance of identifying suitable implementing agencies, funding sources, and addressing systemic barriers such as workforce, policies, and legislation to support the scaling up of mental health interventions during humanitarian crises or generally [32,61,62]. Our study offers a nuanced understanding of perceived factors influencing scalability of psychological intervention within an active war context.

The findings on main barriers for implementing and scaling up CHANGE in war zones are consistent with existing literature [33,34,61,63]. For instance, a systematic literature review highlighted health system factors, and cultural issues such as stigma surrounding mental health as key barriers for scaling up psychological interventions in humanitarian crises [33]. Similarly, Woodward and colleagues [34] identified that a predominantly medicalized approach over preventive strategies, along with conflicting views on provider qualifications and stigma hinder the scalability of psychological interventions for refugees in Jordan. These insights underscore perceived cultural and health system related challenges that need to be addressed to improve scalability and access to mental health support, especially for war affected populations.

Our research draws attention to two interconnected barriers to the scalability of the CHANGE intervention: limited support from potential partners and lack of referrals from primary care services. While state organizations were generally regarded as supportive, the CHANGE programme is currently perceived primarily as a research initiative, still under evaluation and not yet recognized within governmental standards of care. Consequently, it has not been prioritized in official service pathways, which may partly explain limited referrals from primary healthcare providers. Another possible explanation, as indicated by our findings, is that family doctors often face time constraints and heavy workloads, which limit their ability to provide psychosocial referrals. In addition, low help-seeking behaviour among individuals with AUD may further reduce opportunities for referral and engagement.

Another contributing factor is that CHANGE is presently adopted and implemented by non-governmental actors, research institution NaUKMA, and the NGO WordsHelp. Some governmental stakeholders expressed concerns about the long-term sustainability of NGO-led initiatives, viewing them as time-bound and grant-dependent, and raised questions about the professional qualifications, certification, and licensing of psychosocial service providers working within these organizations. In contrast, non-governmental stakeholders, CHANGE implementers and adopters generally described NGOs as flexible, responsive, and well-positioned to reach affected communities, and facilitate referrals. A similar pattern was observed in Jordan, where local NGOs were more open to implementing task-sharing psychological interventions for refugees, while the government sector remained more hesitant [34].

However, this trend appears to be shifting in some countries, as reflected in the recent Jordanian National Mental Health and Substance Use Action Plan, which demonstrates efforts to improve healthcare services and community-based support for refugees [64]. Similarly, Lebanon’s National Mental Health Programme (NMHP), through its 2024–2030 Strategy, promotes community-based care, primary health integration, and task-sharing aligned with WHO mhGAP guidelines to address crisis-driven mental health needs [65]. A parallel trend is emerging in Ukraine, where recent mental health legislative reforms, such as establishment of Coordination Centre of Mental Health [21], and government’s approval of the “Action Plan for 2024–2026 for the Implementation of the Concept of Mental Health Development in Ukraine for the Period up to 2030” [22], reflect increasing political will to mainstream MHPSS programmes, including programmes such as CHANGE. These developments suggest that building cross-sectoral trust and establishing clear quality standards for NGO-delivered services are becoming increasingly recognized as prerequisites for sustainable scale-up. Progress in this direction will likely require continued dialogue between governmental and non-governmental actors around accreditation, licensing, and long-term financing frameworks.

However, stigma surrounding mental health and AUD emerged as a major barrier in our study. Widespread societal stigma coupled with lack of societal awareness and a fear of conscription appear to discourage men in Ukraine from seeking help. This resonates with existing research, where mental health stigma is often cited as a significant barrier to successful implementation and acceptance of psychological interventions [61,66,67]. For example, a qualitative study of Mootz and colleagues [66] found that men frequently perceive alcohol consumption as normal and underestimate its risks, which reinforces stigma and hinders help-seeking behaviour. Another research from Ukraine indicated that alcohol misuse in men occurs within a cultural context where such behaviours are viewed as socially acceptable, with family members sharing similar understandings, as the men themselves [3]. Likewise, studies have demonstrated that mental health stigma can impede scaling up interventions like PM+, due to the belief that only “sick” individuals seek therapy [61,67]. As emphasized in WHO’s Building Back Better approach, addressing stigma through public awareness campaigns and integrating mental health into broader development agendas are critical strategies for establishing sustainable mental health support in humanitarian contexts [68]. Raising public awareness and destigmatizing help-seeking could be essential steps to ensure that conflict-affected populations in Ukraine and beyond can access and benefit from mental health support initiatives such as CHANGE. To further address gender-specific aspects, future research should include more male stakeholders to explore how gender may influence implementation perspectives on stigma and male help-seeking, given that most participants in this study were women.

In addition to the barriers commonly encountered when implementing psychological interventions in peacetime, war introduces unique factors that can threaten the very existence of such programs. Safety concerns leading to missed appointments, limited access to electricity for online participation, fear of conscription, and erosion of social trust, are just a few of the barriers emerged. Similarly, a recent systematic review on addressing the mental health needs during Gaza’s ongoing conflict highlighted major barriers to care, including damaged infrastructure, staff shortages, and economic hardships, all negatively impacting the population’s mental health [69]. Despite the challenges faced in implementing CHANGE, the war context in Ukraine, but also elsewhere [69,70] underscores the increased need for MHPSS programmes that are specifically adapted to war settings. For interventions like CHANGE, such adaptations could also include flexible session scheduling, modular non-sequential formats that allow participants to miss sessions without losing progress, and strengthened homework components focusing on coping strategies.

This research brings to light that effective scale-up of MHPSS interventions in war settings, such as Ukraine, may require a comprehensive approach that aligns with the MHPSS pyramid framework. This pyramid organizes interventions across four layers: basic services and security, community and family support, focused non-specialized support, and specialized clinical services [53,71]. Despite substantial recent efforts to reform Ukraine’s mental health system (e.g., establishment of the MHPSS Technical Working Group [21], the nationwide “How are you?” programme [19], and the adoption of new national action plan [22]), the Lancet Psychiatry Commission on mental health in Ukraine highlights that current implementation efforts still emphasize specialized and clinical services over community-based approaches. Our findings suggest the potential of NGOs and community organizations in implementing MHPSS programmes like CHANGE. However, they also identify challenges, including lack of referrals from primary care, funding constraints and competition among psychological service providers in Ukraine. This competition, marked by service overlap, client confusion, and NGO proliferation since the full-scale invasion, emerged as interconnected perceived barriers, potentially impeding the adoptability of CHANGE.

Implementation science suggests effective implementation frameworks and strategies may support implementing and scaling up health interventions in war settings [45]. Scaling up interventions like CHANGE may require multifaceted efforts focused on stakeholder engagement, continued training and supervision, and community efforts for reducing mental health and AUD stigma and normalizing help-seeking. Participants in our study highlighted the importance of establishing strong collaborations with local communities, authorities, and NGOs to foster support. Similarly, Ndlovu and colleagues [62] emphasize multisectoral integration of MHPSS programmes, identifying adaptivity, funding, social capital, participation and sustainability as key elements. Established partnerships combined with a shared strategic vision appeared to empower the CHANGE team to navigate challenges, such as lack of referrals from primary care and competition among service providers, while advancing implementation. For long-term sustainability, strategies could involve integrating CHANGE into existing health regulations and standards, along with clear provider training, accreditation, and funding models such as municipal budgets and donor advocacy. Additionally, mass media campaigns and frontline services could promote help-seeking and reduce stigma, creating a supportive environment for dissemination and uptake.

Findings from the inner setting and individuals domains collectively highlight workforce development as a critical component of scale-up planning. Team professionalism, contextual expertise, and sustained motivation were perceived as key enablers of implementation quality, while psychological distress and payment instability posed tangible risks to staff retention. In active war contexts, where implementers are simultaneously service providers and affected community members, sustained investment in supervision, psychological support for providers, and stable remuneration must be built into scale-up models from the outset.

Participants’ emphasis on partnerships, policy integration, and sustainable financing suggests potential pathways for system-level scaling beyond pilot delivery. Woodward and colleagues [31] argue that ecological validity requires multilevel embedding of mental health interventions, converting research projects into nationally integrated services. Perceived scalability barriers of CHANGE highlight system-level prerequisites such as coordinated MHPSS delivery across pyramid levels.

This study has several limitations. Although these findings indicate important directions and insights, they remain exploratory in nature. The results should be interpreted with caution, particularly with regard to their implications for large-scale implementation. We conducted all interviews online, which may have impacted the depth of rapport, connection, and trust established between interviewers and participants. Face-to-face interviews might have facilitated better interpretation of non-verbal cues, such as body language. Team members interviewed other members of the CHANGE implementation team, which may have introduced social desirability bias and contributed to the absence of reported barriers within the CFIR *individuals* domain. Furthermore, as all implementers were employed within a well-structured and well-resourced research trial, individual-level barriers that might emerge in less supported implementation contexts may not have been fully captured. Implementation strategies suggested by maintainers may have been influenced by their organizational affiliations, as many represented state organizations, potentially shaping their perspectives on strategy feasibility and priorities. Additionally, although the intervention specifically targets men, most implementers and maintainers interviewed were women, which may have limited the extent to which gendered lived experiences of alcohol use and stigma among men were reflected in the implementation perspectives captured. Not all key stakeholders were able to participate; out of seven invited maintainers, two representatives from governmental organizations declined due to limited availability. War-related stress, insecurity and power dynamics may have also shaped participation willingness and response content. This qualitative study was conducted concurrently with a fully powered Randomized Controlled Trial, evaluating the effectiveness of the CHANGE intervention in Ukraine. As a result, some participants may have viewed the study as premature for assessing the implementation and scalability of CHANGE, which could influence their perceptions. Reassessing the scale-up process at a later stage could provide a clearer understanding of the actual barriers and facilitators involved in scaling up CHANGE in Ukraine.

Despite these limitations, this study presents a robust methodological approach by incorporating perspectives from a diverse range of stakeholders, including program implementers, adopters, and maintainers from both governmental and non-governmental sectors. By triangulating data across these varied perspectives, we achieved a multifaceted understanding of the perceived factors influencing the scalability of the CHANGE intervention in Ukraine, as well as suggested implementation strategies that may facilitate future scale-up. In addition to the CFIR framework [36,37], which provided a well-defined structure for organizing themes across domains, our analysis employed Powell’s discrete implementation strategies framework as outlined in the ERIC study [39,40] further solidifying the study’s methodological rigor. Importantly, throughout the analysis we maintained a deliberate focus on scalability as analytically distinct from implementation. While some barriers identified, such as stigma, referral pathways, and financing constraints, are commonly encountered in implementation research, their significance in this study lies specifically in their system-level implications for scale-up. For instance, stigma is not merely a barrier to individual help-seeking but a population-level deterrent that may limit the reach of CHANGE at scale. Similarly, financing constraints do not only threaten current programme delivery but represent a structural barrier to the public investment required for sustainable population-level coverage. Referral pathways from primary care, if not systematically established through policy integration, would prevent CHANGE from achieving the reach necessary for future scale-up.

Thus, our research provides valuable insights into the implementation and future scaling of brief psychological interventions in ongoing war, contributing to both theoretical understanding and practical applications.

## Conclusion

In light of the persistent mental health disparities and acute challenges in accessing care for the population in war zones, [69,70,72], this exploratory study offers insights into perceived factors influencing scalability of a community-based brief psychological intervention in Ukraine’s war-torn landscape.

From a theoretical perspective, our findings demonstrate that applying CFIR in an active war context reveals a distinct layer of scalability determinants, including provider psychological distress, and safety-driven reluctance to seek help, that extend beyond those documented in stable or post-crisis implementation settings. This suggests that existing implementation frameworks, while valuable, require contextual adaptation when applied in active conflict settings, and that scalability assessments conducted alongside effectiveness trials can generate critical insights for future system-level expansion.

At the policy level, our findings highlight that sustainable scale-up of MHPSS interventions in war contexts is unlikely to be achieved through either NGO-led or government-led approaches alone. Rather, progress will require cross-sectoral dialogue around accreditation, licensing, and long-term financing frameworks, alongside integration of evidence-based programmes into national clinical standards of care. The increasing political will for mental health reform in Ukraine, reflected in recent legislative developments, provides a meaningful foundation for such integration.

In terms of scalability, online delivery adaptation emerged as a core facilitator of implementation resilience in this context, allowing continued programme access despite war-related disruptions. Combined with sustained investment in workforce wellbeing and supervision infrastructure, these adaptations offer practical lessons for scaling psychological interventions in other humanitarian and conflict-affected settings. Findings from this study will directly inform a subsequent Theory of Change workshop, in which stakeholders will collectively develop a structured scale-up pathway for CHANGE. Further research is needed to test the feasibility of identified strategies across implementation phases and to evaluate their effectiveness in supporting multisectoral MHPSS scale-up.

## Supporting information

S1 Appendix CFIR topic guide for CHANGE implementers. (DOCX)

S2 Appendix Coding framework for CHANGE using CFIR. (XLSX)

S3 Appendix Codebook, summary of codes with illustrative quotes. (XLSX)

S4 Appendix Characteristics of study participants. (DOCX)

S5 Appendix Implementation strategies for CHANGE: Identified by stakeholders and organized into ERIC’s Nine Thematic Clusters. (DOCX)
