# Supplementary material for: Stakeholder perspectives on the scalability of a psychological intervention for alcohol misuse and psychological distress in wartime: A qualitative study in Ukraine
Source: PLOS Ment Health. 2026 Jul 7;3(7):e0000639. doi: 10.1371/journal.pmen.0000639 (PMC13340804; doi:10.1371/journal.pmen.0000639)
Supplement: S1 Appendix — (DOCX) [file pmen.0000639.s001.docx]

# **Supporting information**

**S1 Appendix. CFIR Topic Guide for CHANGE implementers** (adapted from Damschroder et al., 2009, doi: 10.1186/1748-5908-4-50; Damschroder et al., 2022, doi: 10.1186/s13012-021-01181-5).

| **OUTER SETTING DOMAIN**  ***Outer Setting:* The setting in which the Inner Setting exists (i.e. Ukraine as a state; the community where the intervention is being implemented).** | | |
| --- | --- | --- |
| **Construct Name** | **Construct Definition *The degree to which:*** | **Question** |
| **Local Attitudes** | Sociocultural values (e.g., shared responsibility in helping recipients) and beliefs (e.g., convictions about the worthiness of recipients) encourage the Outer Setting to support implementation and/or delivery of the innovation. | What types of support were needed from the government/community/NGOs to implement or deliver CHANGE?  What other types of support (if any) would you have liked to receive (that would have helped you in implementing CHANGE)? |
| **Partnerships & Connections** | The Inner Setting is networked with external entities, including referral networks, academic affiliations, and professional organization networks. | To what extent has WordsHelp/NaUKMA established connections (e.g. partnerships and/or contracts) with others to make the implementation and/or delivery of CHANGE easier? E.g. Ministry of Health, NGOs/community-based organizations, hospitals for referrals, etc.  Probe: how have these connections helped make the delivery of CHANGE easier?  What other types of partnerships (if any) would have been helpful to implement CHANGE? |
| **Policies & Laws** | Legislation, regulations, professional group guidelines and recommendations, or accreditation standards support implementation and/or delivery of the innovation. | What rules, standards or guidelines made it easier or harder to implement and deliver CHANGE? (e.g. professional societies, training certificates, Letter of support from NGOs, others)  What political and financial support would help in implementing CHANGE long term? |
| **Societal Pressure** | Mass media campaigns, advocacy groups, or social movements or protests drive implementation and/or delivery of the innovation.  Note: Societal pressures = any social movements that drive implementation, local champions, advocacy groups, etc | Were there any societal movements that influenced implementation and delivery of CHANGE in a positive or negative way? (e.g. locally formed support groups, advocacy groups within the community, others)  Probes: Participants advocating in their communities  What was helpful about them?  What was unhelpful? |
| **INNER SETTING DOMAIN**  **The setting in which the innovation is implemented; i.e. WordsHelp/ NaUKMA as organization; the team within WordsHelp/ NaUKMA)** | | |
| **Construct Name** | **Construct Definition** | **Question** |
| **Work Infrastructure** | Organization of tasks and responsibilities within and between individuals and teams, and general staffing levels, support functional performance of the Inner Setting. | Please describe how general staffing levels in WordsHelp/NaUKMA interacted with, supported or impeded implementation and/or delivery of CHANGE.  Probe: Bureaucracy, reporting structure (power structure), delegation of tasks, schedules, workload (need to work on other interventions), general staffing levels at WordsHelp/NaUKMA, high staff turnover, understaffing |
| **Deliverer-Centeredness (also addresses the incentives domain)** | There are shared values, beliefs, and norms around caring, supporting, and addressing the needs and welfare of deliverers. | To what extent are the needs of the facilitators that deliver  CHANGE understood and addressed by WordsHelp/ NaUKMA? Probe: safety, professional development, work-life balance, salary, holidays, flexibility in planning of caseload? |
| **Materials & Equipment** | Supplies are available to implement and deliver the innovation. | To what extent were necessary materials and equipment (e.g. office equipment, rooms for counselling, power banks, internet access, phones, tablets, materials in the field) available to implement and deliver CHANGE? What would you need for longer maintenance of CHANGE in your setting and who would provide it? |
| **INDIVIDUALS DOMAIN**  ***Individuals:* The roles and characteristics of individuals. [Document the roles applicable to the project and their location in the Inner or Outer Setting.]**  **[Document the characteristics applicable to the roles in the project based on the COM-B system or role-specific theories.]** | | |
| **Construct Name** | **Construct Definition** | **Question** |
| **Implementation Facilitators** | Individuals with subject matter expertise who assist, coach, or support implementation. | Who were implementation facilitators in CHANGE and what was their role? |
| **Opportunity** | The individual(s) has availability, scope, and power to fulfil Role. | How much decision-making power did the facilitators have within the team?  How did this impact on their ability to fulfil their role? |
| **Motivation** | The individual(s) is committed to fulfilling Role. | What motivated the facilitators to join the CHANGE project? Probes: topic, salary/income, personal growth/learning, population group, social significance  What motivated the facilitators to stay engaged? Probes: topic, salary/income, personal growth/learning, population group |
| **Implementation Team Members** | Individuals who collaborate with and support the Implementation Leads to implement the innovation, ideally including Innovation Deliverers and Recipients. |  |
| **Opportunity** | The individual(s) has availability, scope, and power to fulfil Role. | How much decision-making power did the data collectors have within the team?  How did this impact on their ability to fulfil their role? |
| **Motivation** | The individual(s) is committed to fulfilling Role. | What motivated the data collectors to join the CHANGE project and stay engaged? Probes: topic, salary/income, population group, social significance |
|  | *The degree to which individuals:* |  |
| **Tailoring Strategies** | Choose and operationalize implementation strategies to address barriers, leverage facilitators, and fit context. | What were some of the most important implementation challenges you encountered?  How were strategies to address them chosen? (e.g. during supervision, with coordinator, other) |
| **Adapting** | Modify the innovation and/or the Inner Setting for optimal fit and integration into work processes. | How were WordsHelp/NaUKMA work processes adapted to integrate CHANGE into the day-to-day work?  Is there anything that could be done better to implement CHANGE into WordsHelp/ NaUKMA’s long-term working processes? |
| **IMPLEMENTATION OUTCOMES**  **Assessing Anticipated Implementation Outcomes (Outcomes Addendum CFIR)** | | |
| **Outcome** | **Definition** | **Question** |
| **Adoptability** | The likelihood that key decision makers will decide to put CHANGE in place; mental health providers and social workers decide to deliver CHANGE | The successful adoption of a public health intervention means that the target population widely accepts and starts using the intervention.  What is needed to achieve that successful adoption of CHANGE? |
| **Implementability** | The likelihood that CHANGE will be implemented consistently | The successful implementation of a public health intervention means putting the intervention into practice as it was intended, ensuring it is done correctly and reaches the people who need it.  What is needed to achieve that successful implementation of CHANGE?  CHANGE is based on the training of mental health providers and social workers from local communities. What are in your view the challenges when it comes to long-term implementation of CHANGE? How do you think these challenges can be overcome for CHANGE? |
| **Sustainability or Maintenance** | The likelihood that CHANGE will be put in place or delivered over the long-term | The successful sustainment of a public health intervention means keeping the intervention going over time, ensuring it continues to benefit the target population.  What is needed to achieve that successful sustainment of CHANGE?  What do you think are the main obstacles for integrating CHANGE into existing systems including government led health systems and UN/NGO provided services in Ukraine? Prompt: please consider both the government and UN/NGO services in your answer; are there differences we need to consider between the local and national level?  You named certain barriers (repeat what interviewee mentioned). What strategy/ strategies can you think to overcome the barriers that you mentioned? (Note: check for each barrier that was mentioned).  Are there any wider trends which have happened or are currently happening in Ukraine, which could positively or negatively influence the sustainability of the intervention? Probes: Large-scale and/or unanticipated events - War (e.g. Electricity outages, mobile connection issues, military obligations for men), policy changes ((mental)health, work, education, language), economic developments (e.g. financial crises, cuts), socio-cultural developments (within (health) care). |
