# Supplementary material for: Stakeholder perspectives on the scalability of a psychological intervention for alcohol misuse and psychological distress in wartime: A qualitative study in Ukraine
Source: PLOS Ment Health. 2026 Jul 7;3(7):e0000639. doi: 10.1371/journal.pmen.0000639 (PMC13340804; doi:10.1371/journal.pmen.0000639)
Supplement: S4 Appendix — (DOCX) [file pmen.0000639.s004.docx]

# **Supporting information**

**S4 Appendix. Characteristics of Study participants**

| **N** | **Role** | **Gender** | **language of the interview** | **Location (East, North, West, South, Central Ukraine)** | **Organization** |
| --- | --- | --- | --- | --- | --- |
|  | **Implementer** Data collector | Female | English | Dnipro, East | CHANGE project team - NaUKMA Mental Health Center /WordsHelp |
|  |  | Female | Ukrainian | Kyiv, Central |  |
|  |  | Female | Ukrainian | Dnipro, East |  |
|  | **Implementer** Facilitator | Female | Ukrainian | Kyiv, Central |  |
|  |  | Female | Ukrainian | Dnipro, East |  |
|  |  | Female | Ukrainian | Kyiv, Central |  |
|  |  | Female | Ukrainian | Kyiv, Central |  |
|  |  | Female | Ukrainian | Vinnitsa, Central |  |
|  |  | Female | Ukrainian | Poltava, Central |  |
|  | **Implementer** Project manager | Female | English | Kyiv, Central |  |
|  | **Implementer** Supervisor | Female | English | Dnipro, East |  |
|  |  | Male | Ukrainian | Kyiv, Central |  |
|  | **Implementer** Recruitment officer | Female | Ukrainian | Kyiv, Central |  |
|  | **Adaptor** | Male | English | Kyiv, Central  *(before the war, currently abroad)* | NaUKMA Mental Health Center /WordsHelp |
|  |  | Female | Ukrainian | Kyiv, Central |  |
|  | **Maintainer** | Female | Ukrainian | Kyiv, Central | Public Health Centre of the Ministry of Health (MoH) |
|  |  | Male | English | Kyiv, Central | United Nations Office on Drugs and Crime (UNODC), MoH |
|  |  | Male | Ukrainian | Kyiv, Central | Ukrainian Red Cross, formally Ministry of Veterans Affairs |
|  |  | Female | Ukrainian | Kyiv, Central | Governmental (state) initiatives: HOW ARE YOU? |
|  |  | Female | Ukrainian | Kyiv, Central | Kyiv City Center for Social Services |
| ***Total N 20*** *(13 implementers, 2 adopters, 5 maintainers)* | | | | | |
