# Supplementary material for: Stakeholder perspectives on the scalability of a psychological intervention for alcohol misuse and psychological distress in wartime: A qualitative study in Ukraine
Source: PLOS Ment Health. 2026 Jul 7;3(7):e0000639. doi: 10.1371/journal.pmen.0000639 (PMC13340804; doi:10.1371/journal.pmen.0000639)
Supplement: S5 Appendix — (DOCX) [file pmen.0000639.s005.docx]

# **Supporting information**

**S5 Appendix. Implementation Strategies for CHANGE: Identified by Stakeholders and Organized into ERIC’s Nine Thematic Clusters** (cluster and discrete strategy names adapted from Powell et al., 2015, doi: 10.1186/s13012-015-0209-1; Kirchner et al., 2020, doi: 10.1016/j.psychres.2019.06.042).

| **N** | **Cluster** | **Discrete strategy** | **CHANGE - strategy identified by stakeholders** |
| --- | --- | --- | --- |
|  | **Use evaluative and iterative** | Audit and provide feedback | - Conduct ongoing monitoring and evaluation |
|  |  | Purposefully re-examine the implementation | - Deliver intervention remotely (decision made based on pilot study results, showing challenges in implementing the intervention face-to-face) |
|  |  | Conduct local need assessment | - Conduct needs assessment in communities |
|  |  | Obtain and use patients/consumers and family feedback | - Engage with communities and receive their feedback |
|  | **Provide interactive assistance** | Facilitation | - Make intervention delivery flexible; - Provide implementation team an opportunity of a continuous supervision and support. |
|  |  | Provide local technical assistance | - Get help from community to recruit participants - Partner with local organizations who can support training some providers |
|  | **Adapt and tailor to context** | Tailor strategies | - Connect with existing local or national wide programs and put efforts in integrating CHANGE into their platforms |
|  |  | Promote adaptability | - Adapt the intervention to military personnel or men who are affected by the war |
|  | **Develop Stakeholder interrelationships** | Identify and prepare champions | - Connect and get support from representatives of regional administrations to show that programme is supported from some state agencies - Recruit consultants in the communities who can promote and support CHANGE activities |
|  |  | Recruit, designate, and train for leadership | - Recruit consultants in the communities |
|  |  | Build a coalition | - Partner with NGOs who already have social workers - Map organizations working on similar issues - Create a network of specialists - Create a collaborative environment with service providers to develop a system in which they are not competitors but instead, they support each other - Engage faith-based and community organizations (Religious organizations) - Partner with organizations providing integrated services |
|  |  | Obtain formal commitment | - Make a formal agreement e.g., memorandum with organizations who will support CHANGE |
|  |  | Use advisory boards and workgroups | - Conduct discussions, e.g., focus group discussions to learn from field experts and providers, (other organizations providing similar services) |
|  |  | Involve executive boards | - Connect with governmental institutions, and work together to integrate intervention like CHANGE into their regulatory frameworks |
|  |  | Develop academic partnership | - Partner with educational institutions who deliver trainings and who can train CHANGE implementation team in future |
|  | **Train and educate stakeholders** | Conduct ongoing training | - Decide who will provide training certificates |
|  |  | Develop educational materials | - Provide information materials - Educate the population in war about aim of CHANGE |
|  |  | Conduct educational meetings | - Integrate CHANGE into certificate program – e.g., degree programs - Ensure continues supervision |
|  |  | Create learning collaborative | - Partner with organizations who provide training to develop and deliver training using their support |
|  |  | Work with educational institutions | - Partner with educational institutions who can support in training facilitators |
|  | **Support clinicians** |  | - Not applicable |
|  | **Engage consumers** | Involve patients/consumers and family members | - Involve participants and their family members who benefited from CHANGE to promote the programme in communities |
|  |  | Prepare patients/consumers to be active participants | - Work with participants to encourage them to stay in the program, build trust in community and educate them about the benefits of CHANGE |
|  |  | Increase demand | - Communicate the effectiveness of CHANGE, show it as evidence-based intervention - Disseminate information though front-line professionals and family doctors - Launch a concise and action-oriented campaign, simple advertising, including commercials on various platforms (YouTube, public transportation, billboards with self-assessment questions, etc); - Initiate/engage in national campaigns focused on male emotions and needs, addressing alcohol misuse stigma; - Raise awareness about topic of addictions. - Focus on public organizations, local authorities, businesses, military for reaching more men |
|  |  | Use mass media | - Conduct information campaigns using social media channels e.g., Facebook, Telegram, YouTube, etc. |
|  | **Utilize financial strategy** | Access new funding | - Get financial support from international organizations e.g., WHO Office Ukraine - Attract donor funds to support training and service provision - Advocate with municipal authorities to take responsibility of co-financing CHANGE |
|  | **Change infrastructure** | Change record systems | - Create data tracking platform for specialists and participants |
|  |  | Change service sites | - Make intervention delivery flexible |
