# Supplementary material for: Stakeholder perspectives on the scalability of a psychological intervention for alcohol misuse and psychological distress in wartime: A qualitative study in Ukraine
Source: PLOS Ment Health. 2026 Jul 7;3(7):e0000639. doi: 10.1371/journal.pmen.0000639 (PMC13340804; doi:10.1371/journal.pmen.0000639)
Supplement: Attachment — Submitted filename: Response to Reviewers.pdf [file pmen.0000639.s007.pdf]

Response to Reviewers – Manuscript ID: PMEN-D-25-00606

Scalability of a psychological intervention for alcohol misuse and psychological distress in wartime: A qualitative study in Ukraine

PLOS Mental Health

Dear Mr. Shishir Paudel, and Reviewers,

We would like to sincerely thank you for the thoughtful and constructive feedback on our manuscript. We greatly appreciate the time and effort that you and the reviewers dedicated to providing detailed suggestions, which have helped us to substantially improve the clarity and quality of the paper.

Below, we provide a detailed, point-by-point response to each comment raised by the editor and reviewers. Our responses are presented in a table format. All corresponding changes have been made in the revised manuscript and are highlighted using track changes. For each revision, we have also indicated the relevant page and line numbers where the modifications appear.

We hope that the revised version of the manuscript now meets the expectations of the editor and reviewers and will be considered suitable for publication in PLOS Mental Health.

Thank you again for your time and consideration.

With kind regards,

Dr. Khatia Antia

on behalf of all co-authors

Leibniz Institute for Prevention Research and Epidemiology, BIPS

antia@leibniz-bips.de

Journal Requirements:	Authors' response
1. Please ensure that your Ethics Statement is available in its entirety at the beginning of your Methods section, under a subheading 'Ethics Statement'.	We have revised the manuscript accordingly and moved the Ethics Statement sub-section to the beginning of the Methods section, directly below the Materials and Methods heading, as requested.<br><br>***Page 6, lines 137-149***
2. We note that you have indicated that there are restrictions to data sharing for this study. For studies involving human research participant data or other sensitive data, we encourage authors to share de-identified or anonymized data. However, when data cannot be publicly shared for ethical reasons, we allow authors to make their data sets available upon request. For information on unacceptable data access restrictions, please see http://journals.plos.org/plosone/s/data-availability#loc-unacceptable-data-access-restrictions.<br>Before we proceed with your manuscript, please address the following prompts:<br><br>a) If there are ethical or legal restrictions on sharing a de-identified data set, please explain them in detail (e.g., data contain potentially identifying or sensitive patient information, data are owned by a third-party organization, etc.) and who has imposed them (e.g., a Research Ethics Committee or Institutional Review Board, etc.). Please also provide contact information for a data access committee, ethics committee, or other institutional body to which data requests may be sent.<br>b) If there are no restrictions, please upload the minimal anonymized data set necessary to replicate your study findings to a stable, public repository and provide us with the relevant URLs, DOIs, or accession numbers. Please see http://www.bmj.com/content/340/bmj.c181.long for guidelines on how to de-identify and prepare clinical data for publication. For a list of recommended repositories, please see https://journals.plos.org/plosone/s/recomm	Thank you for this important guidance on data availability.<br><br>Ethical restrictions (prompt a): This qualitative study involved 20 in-depth interviews with key informants in Ukraine's small mental health implementation field (2 adopters, 13 implementers including project manager, 5 maintainers from governmental/international entities). Full transcripts, even de-identified, reveal unique combinations of organizational roles, and institutional positions that may risk participant identification.<br><br>These restrictions were imposed by:<br><br>- National University of Kyiv-Mohyla Academy (NaUKMA) Ethics Committee (Approval #FWA00030125)<br><br>Data requests should be directed to:<br>NaUKMA Institutional Review Board (IRB) Chairperson – Dr. Tetiana Yurochko, t.yurochko@ukma.edu.ua<br><br>Data transparency measures (prompt b): While full transcripts cannot be shared, we have uploaded as Supporting Information:<br><br>- S2: Codebook (thematic codes and definitions using CFIR)<br><br>- S3: Minimal de-identified dataset (codebook with code summaries and illustrative quotes, excluding identifiable details)<br><br>See Methods section ***Page 10, Lines 237-238***<br><br>We have updated Data Availability Statement.

ended-repositories. You also have the option of uploading the data as Supporting Information files, but we would recommend depositing data directly to a data repository if possible.	
If the reviewer comments include a recommendation to cite specific previously published works, please review and evaluate these publications to determine whether they are relevant and should be cited. There is no requirement to cite these works unless the editor has indicated otherwise.	Thank you for this clarification. While the reviewers did not recommend specific sources, they suggested that we engage more with the international literature on research conducted in war-affected contexts. In response, we have incorporated additional relevant citations throughout the manuscript to strengthen the contextualization of our findings. All new references are included in the revised version and highlighted using track changes.
**Additional Editor Comments (if provided):**	
Please focus on methodological transparency as suggested by the Reviewers.	Thank you for this helpful guidance. We have carefully addressed methodological transparency as recommended by the reviewers. All aspects raised regarding methodological detail and clarity have been responded to in the table below and incorporated into the revised manuscript. Additionally, we uploaded codebook and summary or results/codes as supplementary materials.<br><br>The corresponding changes are highlighted in the document using track changes and specified with page and line numbers.
**Reviewer comment**	**Authors' response**
**Reviewer 1**	
Thank you to the authors and the PLOS editorial team for giving me the opportunity to review this vital and timely manuscript; I believe it contributes significantly to the field of war and emergency mental health interventions, particularly given the ongoing situation in Ukraine.<br><br>Firstly, I'd like to commend the decision to adapt a robustly tried and tested MH intervention, the WHO's problem management plus programme and mhGAP, for alcohol misuse in an active war zone. Second, the overall qualitative findings lend well to wider implementation of the CHANGE intervention as the study interviews a broad range of stakeholders involved in the delivery,	We thank the reviewer for their thoughtful and encouraging feedback. We truly appreciate the recognition of the relevance of our work to the field of war and emergency mental health interventions.<br><br>We have carefully addressed each of the points raised in the revised version of the manuscript. The corresponding changes are highlighted in the manuscript and indicated with page and line numbers in our detailed responses below.

implementation, and reception of the intervention.<br><br>I have highlighted a few suggestions and edits below:	
**1. Introduction section:**	
- Lines 59 - 60; you can also consider drawing on other conflict-affected populations here that might be more relevant to war activities; for example, the American opioid crisis and its particular effects on the American veteran population.	To address this while maintaining focus on alcohol misuse among war-affected populations, we have revised lines 59–60 and incorporated findings from systematic reviews confirming these elevated risks and patterns in displaced groups.<br><br>***Page 3 lines 58-62***
- Line 78 - 79; would it be possible to give the measurements for alcohol use in units? Units are widely used for reporting alcohol consumption in the UK, US and so on, which is where I assume a majority of PLOS MH's readership is located.	We have added standard unit equivalents (~1.5 units for men; ~0.3 for women) alongside the original WHO-reported grams.<br><br>***Page 3-line 68***
- Line 80; as this study sought to adapt and deliver this intervention to men, it is advisable in this section of the introduction to provide a bit more context as to why alcohol consumption as a coping strategy is more widespread/prevalent in men compared to women. I believe this is an important premise to provide in order to justify the subsequent sampling decisions.	We thank the reviewer for this key suggestion to contextualize gender differences in alcohol coping, strengthening the rationale for our male-focused intervention. We have expanded the paragraph.<br><br>***Page 3, line 69***
- Line 120 - 128; I agree with the decision to use the CFIR framework; I believe it is well suited to determining the implementation barriers and facilitators of CHANGE in the context of war in Ukraine. However, I am wondering if it would be possible to give an example of a previous study where the CFIR was used in war or similar contexts? this would help with contextualising the on-the ground reality of conducting a CFIR evaluation.	We thank the reviewer for highlighting this aspect. we have included examples of prior studies applying CFIR (and complementary ERIC strategies) in similar settings, such as refugee interventions (e.g., displaced, migrant and host community women in Latin America), resource-limited environments, and conflict-settings. These are cited in the revised paragraph.<br><br>***Page 5, lines 113-119***
- Line 134-143; this paragraph includes information about the intervention's methodology and delivery and therefore would be better placed into the study's materials and methods section.	We thank the reviewer for this suggestion. We have moved the paragraph describing the intervention's methodology and delivery to the Methods section.<br><br>***Pages 6, lines 150-165***
**2. Materials and methods;**	
- Line 155; I would recommend splitting up this section into two; one section being the study design, and second being the sample. For the sample section, it might be helpful to include information about who (from which org, in what capacity) was interviewed, and for the study	We have split the section into "Study Design" and "Participants" subsections, added rationale for each stakeholder category, and included organizational/capacity details in the Participants subsection.

design section to provide information on why (for example, 'adopters' were needed to understand the extent and features of integrating CHANGE into pre-existing practices). Nevertheless, this can be considered a stylistic choice, so feel free to amend or disregard as you see fit.	***Pages 7-8, lines 166-198***
- Line 209; was the same certified translator used for all the interviews that needed translating? this is useful information to provide as using a variety of translators from the same service might result in differences in how certain text or non-text cues might be interpreted. Perhaps something to consider highlighting in the discussion section as well.	We thank the reviewer for this important clarification request. We have specified that all 15 Ukrainian transcripts were translated by the same certified translator.<br><br>***Page 9, lines 217-220;***
- Line 217; would it be possible to provide a bit more information about how the research team collaborated in thematically analysing the findings? for example, if any researcher reflexivity practices were followed, if there was a first and second coder, how collective decisions were made regarding top level codes, validity checks, etc. This information, if made available, can greatly boost the perceived validity of your findings	We thank the reviewer for this valuable suggestion to enhance methodological transparency. We have expanded section to detail the data analysis and added a reflexivity statement describing researchers ' role in data collection and their positionality.<br><br>***Page 10, lines 228-231***
**3. Results and Discussion section:**	
- Line 235; I assume this means a majority of the interviewees apart from the stakeholders were female? As the CHANGE programme is designed to specifically address excessive alcohol consumption in men, it would be helpful for the study's discussion section to identify this as a limitation as one could argue certain lived experience aspects of being a male might be useful to understanding the barriers and facilitators of implementing CHANGE. i.e, when it comes to assumptions about uptake, how to address stigma, etc.	We thank the reviewer for highlighting this important point. We have added a sentence in the limitations noting that most implementers and maintainers interviewed were women, potentially limiting direct insights into male lived experiences of alcohol use and stigma.<br><br>***Page 30, lines 616-618***
- Table 1; Construct names 'Culture' and 'Available resources'; how is psychological distress amongst the CHANGE implementation team handled? Would be helpful for the methods section to include some detail about the supervision and/or pastoral support provided to implementers of CHANGE.	We thank the reviewer for this helpful suggestion. We have added the paragraph to the Methods section to address psychological distress support and supervision for the CHANGE team, corresponding to Table 1 constructs 'Culture' and 'Available resources':<br><br>***Page 6, lines 158-163***
- Line 421 to 423 and line 627 to 628; is it possible to provide some contextual information about what other intervention offerings are available? For example, include in the introduction section which organisations are offering similar or comparable psychosocial	We thank the reviewer for this helpful suggestion to provide additional MHPSS context. Following overall reviewer feedback to make the Introduction more concise, we have retained the existing overview (***Page 3, lines 72-81***) covering Ukraine's key programs

interventions and the characteristics of these interventions. That would be helpful to understanding the premise of this section.	and coordination mechanisms (e.g., WHO, TWG partners, nationwide "How are you? programme"), which already highlights the scarcity of alcohol-focused interventions like CHANGE.
- Line 459 to 464; as the recipients of CHANGE were identified in the introduction section to be largely internally displaced populations, I am wondering if it might be useful to provide information in the discussion section on how the CHANGE programme can be adapted further to account for the frequent disruptions to sessions that recipients might encounter. A few examples could be; increased flexibility on the part of treatment providers in the sessions, making the sessions independent and not consecutive so there is no knock-on impact from missing a session or two, flexible delivery formats, more emphasis on homework tasks so longitudinal progress of the recipient is retained, etc.	We thank the reviewer for these insightful adaptation suggestions. We have revised lines to emphasize the need for war-adapted MHPSS programs, with specific examples for CHANGE (flexible scheduling, modular formats, enhanced alcohol-focused homework).<br><br>(***Pages 28-29, lines 569-571***)
**Discussion**	
- Line 590; stigma from accessing family or community-based MH interventions has been identified as a significant barrier by stakeholders in this study; I imagine a proportion of this stigma also arises from the prevalent gender roles and expectations for how men are 'expected' to cope with adversity compared to women. nevertheless, as the majority of the stakeholders interviewed in this study identified as female, it would be helpful to add a recommendation here for future research into CHANGE to be conducted with male stakeholders to understand if/how gender can impact uptake/effectiveness/acceptability of the intervention.	We appreciate the reviewer highlighting this important gender-stigma connection. We have incorporated this perspective and added a recommendation for future research with more male stakeholders.<br><br>(***Page 28, lines 556-559***)
**Reviewer #2:**	
This is an important and timely piece of research that explores the role of AUD in a specific traumatised population. I would like to see a little more detail in respect of “scaling up”. The premise of the study is to look at how tenable scaling up to war settings (lines 144 onwards) however I feel it needs a little more exploration in the introduction. Maybe even a little more discussion of ecological validity of such treatment programs.<br>The data is as expected and its important to look at these problems in the context of a real world application.	We thank the reviewer for their positive assessment and suggestions regarding “scaling up” and ecological validity. We have made the following revisions.<br><br>**The Introduction** (lines 144 onwards in original submission) has been revised to clarify the study's scalability focus, alongside the existing scaling definition and framework rationale. These changes also respond to similar comments from other reviewers.<br><br>***Pages 4-5, lines 90-119***

I would like to note that I enjoyed reviewing this manuscript.	We strengthened scaling focus across manuscript: results explicitly frame findings through scalability lens;<br><br>We have added a paragraph in the Discussion section addressing the ecological validity and scaling up concepts applying Woodward et al.'s (2021) system innovation framework to highlight multilevel embedding requirements for scaling psychological interventions from pilot delivery to nationally integrated services, in line with our study findings.<br><br>***Page 30, lines 601-605***
**Introduction**	
The introduction outlines the problem in its wider context and speaks to the issues in delivering a successful intervention-based treatment program. I might suggest that an overview paragraph is included at the beginning of this section before the authors begin to outline the issues to be addressed.	We thank the reviewer for this helpful suggestion. We have revised the entire Introduction section to better outline the problem within its wider context and clearly articulate the issues in delivering intervention-based treatment programs in wartime settings.<br><br>This revision also incorporates feedback from multiple reviewers.<br><br>(***Pages 3-6, lines 55-135***)
**Methods**	
This section describes a classic thematic analysis approach. Attention to ethical detail especially in respect to enhanced approach to consent to use quotes is commendable.<br>While it seems that every attempt has been made to reduce subjective bias in the coding of the data would it have been helpful to guide research team discussions around the themes based on multiple coding from the team?	We appreciate the reviewer’s positive comment on our ethics procedures.<br><br>The comment regarding research team discussion and coding also aligns with Reviewer 1’s feedback on analysis transparency, which we have addressed by expanding methods section to detail the coding process and team discussions leading to final themes and sub-themes. We also added supplementary materials – a codebook, and a summary codes/results and cited them in the methods section.<br><br>(***Page 10, lines 234-244***)
**Results**	
The data is compelling and clearly identifies a disconnect withing the context of the scalability of the program to be effective. The tables provide a nice overview of the data. As with all thematic analysis it is often hard to disentangle the interpretive nature of the data. However, there is a well-defined framework with the results	We appreciate the reviewer's positive assessment of the compelling data. To address the interpretive nature of thematic analysis and make the well-defined framework more explicit, we have:<br><br>Revised the Results section introductory paragraph to emphasize deductive

section. This could have been made a little more explicit to strengthen the data presented.	organization by CFIR domains alongside emergent themes ***(page 11, lines 264–266)***;<br><br>Updated Table 1 title to 'Summary of Perceived Implementation Barriers and Facilitators in the CHANGE Implementation, Organized by CFIR Domains' ***(page 13, line 271);*** and<br><br>Added explicit reference to CFIR's structuring role within the Discussion's methodological strengths paragraph ***(page 31, lines 632–634)***
**Discussion**	
One suggestion that is probably one of those reviewers' comments that are aligned with "but we didn't study that", might be useful for future work is to look at this within the theoretical framework of Activity Theory. I think it might give a nice framework to try and look at how different stakeholders could be aligned.	We thank the reviewer for this helpful suggestion on stakeholder alignment. We've added a recommendation to the Conclusion "We recommend investigating conceptual factors and implementation strategies across implementation phases, alongside multisectoral coordination for MHPSS scale-up. ***(page 32, lines 649-651)***.<br><br>We kept it general rather than naming Activity Theory specifically, as our study is framed around CFIR and ERIC, and introducing a new theoretical lens could undermine the primary focus without direct empirical links in our data.
**Reviewer #3:**	
This manuscript addresses a highly relevant and timely topic, namely the scalability of a psychological intervention for alcohol misuse and psychological distress in the context of active war in Ukraine. The study has clear potential to contribute to the global mental health and implementation science literature, particularly for humanitarian and conflict-affected settings. However, while the topic and overall approach are appropriate for PLOS Mental Health, several methodological, analytical, and reporting issues currently limit the strength of the evidence and the validity of the conclusions. Substantial revision is required to improve clarity, rigor, and alignment between the data presented and the claims made.	We appreciate the constructive feedback regarding methodological, analytical, and reporting issues and have undertaken substantial revisions to enhance clarity, rigor, and evidence-conclusion alignment throughout the manuscript. The corresponding changes are highlighted in the manuscript and indicated with page and line numbers in our detailed responses below.
**Title and abstract**	
With respect to the title and abstract (pp. 1–2), the manuscript frames the study as an assessment of "scalability," yet the data primarily capture stakeholder perceptions and expectations rather than empirical evidence of	We thank Reviewer 3 for highlighting potential conceptual overstatement.<br><br>We have revised the title to 'Stakeholder Perspectives on the Scalability of the CHANGE

scale-up feasibility or effectiveness. This creates a degree of conceptual overstatement, particularly in the abstract, where conclusions imply readiness for scale-up. The authors are encouraged to refine the title and abstract to more clearly reflect the exploratory and perception-based nature of the findings, and to explicitly distinguish between perceived facilitators and barriers and demonstrated scalability outcomes.	Intervention in Wartime Ukraine' and modified the abstract to emphasize stakeholder perceptions of scalability rather than empirical scale-up outcomes.<br><br>We have also added the following sentence to the abstract: "Here scalability refers to potential scale up of CHANGE, rather than retrospective evaluation of actual scale-up, feasibility or effectiveness."<br><br>***Page 2. Lines 31-33, 37, 44.***
**Introduction**	
The introduction (pp. 3–5) provides a comprehensive overview of alcohol misuse, psychological distress, and the Ukrainian wartime context, but it is at times overly detailed and repetitive. While the epidemiological background is strong, the specific knowledge gap addressed by this study could be articulated more sharply. The authors would strengthen this section by streamlining background content, reducing repetition, and more clearly positioning the study within existing literature on implementation and scale-up of mental health and psychosocial support interventions in humanitarian settings. Explicit justification for the use of qualitative methods to address the stated research questions would also improve conceptual coherence.	We thank the reviewer for this helpful comment and have revised the Introduction accordingly. We streamlined the background to reduce repetition and focused more clearly on the implementation and scale-up of MHPSS interventions in humanitarian settings.<br><br>***Page 3-6. Lines 55-135.***
The use of the Consolidated Framework for Implementation Research and the ERIC implementation strategies framework (pp. 5–6) is appropriate; however, the rationale for selecting these frameworks is presented descriptively rather than critically. The manuscript would benefit from a clearer explanation of why CFIR and ERIC are particularly suitable for examining scalability in wartime or humanitarian contexts, and how they compare to alternative implementation frameworks. Greater clarity on how the study's research questions map onto specific CFIR domains would also enhance transparency and conceptual alignment.	We thank the reviewer for this valuable feedback. In the Introduction, we now explain why CFIR and ERIC are particularly suited to wartime contexts, and clarify how research questions map onto CFIR domains. ERIC is now also introduced as complementary to CFIR.<br><br>***Page 5-6. Lines -103-119, 130-133***
**Methods**	
In the Methods section describing study design and sampling (pp. 6–7), the qualitative approach is appropriate, but key methodological details are insufficiently reported. The manuscript does not clearly justify the sample size, nor does it explain how thematic saturation was assessed or	We thank the reviewer for highlighting these methodological details. We have now clarified our targeted purposive sampling approach in Methods, explaining that sampling prioritized maximum variation across specific roles rather than thematic saturation, consistent with key

whether saturation was a guiding principle. Additionally, the implications of recruiting participants primarily from implementing and policy-related organizations are not fully addressed. The authors should clarify how participant roles and institutional affiliations may have shaped responses, and more explicitly acknowledge any limitations this introduces for generalizability and interpretation.	informant approaches in implementation science.<br><br>***Page 8. Lines 180-183***<br><br>We have addressed the implications of recruiting primarily from implementing and policy organizations by noting in the Limitations section.<br><br>***Page 30. Lines 613-615***
Regarding data collection procedures (pp. 7–8), the authors provide a basic description of interview logistics, consent, and translation processes. However, there is limited reflection on the challenges of conducting qualitative research during active conflict, including how war-related stress, insecurity, or power dynamics may have influenced participation and responses. The authors are encouraged to include a brief reflective discussion on these issues and to further clarify how translation accuracy and meaning equivalence were ensured during transcription and analysis.	We have added to Methods details about data collection during active war (including power outage rescheduling) and clarified that translation accuracy was verified by the third author (native Ukrainian speaker who conducted Ukrainian interviews).<br><br>***Page 10. Lines 224-231***<br><br>We thank the reviewer for this important point. Potential influences of war-related insecurity and power dynamics on participation and responses are now addressed in Limitations.<br><br>***Page 30. Lines 620-621***
The data analysis section (pp. 8–9) lacks sufficient detail to fully assess analytic rigor. While the use of thematic analysis guided by CFIR is stated, the manuscript does not clearly specify whether a reflexive or code-reliability approach was employed, nor how analytic decisions were documented and validated. To strengthen trustworthiness, the authors should elaborate on steps taken to enhance rigor, such as team-based interpretation, audit trails, or reflexive discussion. A brief reflexivity statement acknowledging the researchers' positionality and potential interpretive influences would further strengthen the analysis.	We thank the reviewer for highlighting analytic rigor concerns. We have revised data analysis section to clarify these details.<br><br>***Page 10. Lines 223-232***<br><br>A reflexivity statement has been added acknowledging researchers' positionality and potential interpretive influences.<br><br>***Page 10. Lines 234-244***
**Results**	
In the Results section (pp. 9–13), the findings are generally well organized according to CFIR domains, but several themes overlap conceptually, leading to redundancy. In addition, some sections remain largely descriptive, with limited interpretive depth. While participant quotations are valuable, they occasionally dominate without sufficient analytical synthesis. The authors are encouraged to consolidate	We thank the reviewer for the valuable feedback on the Results section. The results section is now revised to address concerns about theme overlap, descriptive content, and quote integration:<br><br>- Related barriers and facilitators have been consolidated within each CFIR domain

overlapping themes, strengthen interpretive commentary explaining why identified barriers and facilitators matter for scalability, and ensure that quotations consistently support clear analytic points rather than standing alone as illustrative material.	- All findings are now explicitly framed as stakeholder perceptions from qualitative interviews<br>- Participant quotes have been shortened and directly linked to analytic points.<br><br>***Pages 15-25***
**Discussion**	
The Discussion section (pp. 13–16) appropriately situates the findings within the broader literature, but at times overextends conclusions beyond what the qualitative data can support. In particular, claims regarding scalability and policy relevance should be more cautiously framed, given that the study captures perceptions rather than tested scale-up outcomes. The discussion would benefit from clearer differentiation between implications for future research, considerations for implementation planning, and broader policy relevance. Stronger engagement with international literature on task-sharing and humanitarian mental health would also enhance the contribution.	We thank the reviewer for highlighting the need for cautious framing in the Discussion.<br><br>Throughout ***pages 25-31***, we systematically tempered scalability and policy claims by consistently framing findings as exploratory qualitative insights rather than tested outcomes, using precise implementation science language to distinguish stakeholder perspectives, future research implications, implementation planning considerations, and policy relevance.<br><br>We also strengthened Limitations cautioning against large-scale implementation<br><br>***Page 30, Lines 606-608***
Ethical considerations and data availability (pp. 8, 16) are addressed, but somewhat superficially given the sensitivity of conducting research in a wartime context. The manuscript would be strengthened by deeper reflection on ethical challenges such as participant vulnerability, informed consent under duress, and researcher responsibility. In addition, while restrictions on data sharing are understandable, the Data Availability Statement should more clearly specify what de-identified materials (e.g., codebooks or analytic summaries) could be made available upon reasonable request, in line with PLOS data transparency expectations.	We appreciate the reviewer highlighting the need for deeper ethical reflection in wartime research.<br><br>To address this, we expanded the Methods section ***(page 7, lines 158-163)*** with details including well-being and support provided to implementers who participated in this study.<br><br>Also, procedures related to conducting interviews via Zoom given active war (***page 10, lines 223-232).*** We also added a reflexivity statement addressing positionality and power dynamics.<br><br>We updated the Data Availability Statement and made the codebook and summary of codes/results available as supplementary materials. We also added contact details for the NaUKMA Institutional Review Board (IRB), to whom data requests should be directed.<br><br>Please also see our responses to the journal requirements above.
**Conclusion**	

Finally, the conclusion (p. 16) largely reiterates the study's findings but does not sufficiently temper claims regarding scalability. The authors are encouraged to reframe the conclusion to emphasize the study's exploratory contribution to understanding perceived barriers and facilitators, rather than implying readiness for immediate large-scale implementation. Clearly positioning the findings as hypothesis-generating would better align conclusions with the qualitative evidence presented.	We thank the reviewer for this valuable point. We would like to clarify that we do not intend to imply readiness for immediate large-scale implementation. We have carefully reviewed and revised the manuscript to ensure that such an interpretation is avoided.<br><br>***Pages 31-32 Lines 640-651***<br><br>Furthermore, we have expanded the limitations section to emphasize that, although the findings indicate important directions and insights, they remain exploratory in nature. Accordingly, the results should be interpreted with caution, particularly with regard to their implications for large-scale implementation.<br><br>***Pages 30 Lines 606-608***
In summary, this manuscript addresses an important and under-researched topic with significant potential relevance for global mental health practice in conflict settings. However, major revisions are required to improve methodological transparency, analytical rigor, and alignment between findings and conclusions. Addressing the issues outlined above would substantially strengthen the manuscript and its suitability for publication in PLOS Mental Health.	We sincerely thank the reviewer for recognizing the importance of our study for global mental health in conflict settings.<br><br>We have addressed all concerns raised about methodological transparency, analytical rigor, and alignment between findings and conclusions. We hope these revisions have substantially strengthened the manuscript.
**Reviewer #4:**	
This manuscript addresses a timely and important topic: the scalability of a psychological intervention for alcohol misuse and psychological distress during active wartime in Ukraine. The use of CFIR and ERIC frameworks, along with perspectives from implementers, adopters, and maintainers, is a clear strength. The paper offers valuable contextual insights into how war-related disruptions, stigma, funding instability, and NGO–government dynamics shape implementation. However, several areas require strengthening before the manuscript is suitable for publication.	We sincerely thank Reviewer for acknowledging the timeliness and importance of our study, as well as the strengths of our CFIR/ERIC framework application. We have made substantial revisions throughout the manuscript to address the areas identified for strengthening (detailed below with specific page and line numbers). We hope these changes now make the paper suitable for publication.

First, the concept of "scalability" needs clearer differentiation from general implementation, feasibility, or sustainability. While barriers and facilitators are well described, it is not always clear how these specifically relate to scale-up beyond routine delivery. Clarifying what scale would concretely mean in this context (e.g., national integration, public financing, primary care embedding) would strengthen the contribution.	We thank the reviewer for this feedback, also raised by other reviewers. We have clarified scalability throughout:<br><br>**Introduction:** now defines scaling as system-level institutionalization (national policy, public financing, primary care embedding).<br><br>***Pages 4-6, lines 90-135***<br><br>**Results:** barriers are now framed as scalability constraints (detailed in Reviewer 3 response)<br>***Pages 15- 17***<br><br>**Discussion:** we added a paragraph discussing potential pathways for system-level scaling beyond pilot delivery.<br><br>***Page 30, lines 601-605***
Second, the analysis is largely descriptive and closely follows CFIR domains. Greater interpretive depth would improve the manuscript. For example, tensions between NGOs and governmental actors, concerns about professional legitimacy, competition among providers, and stigma in the context of masculinity and mobilization fears could be more critically examined. Moving beyond categorization toward theoretical insight would elevate the paper.	We thank Reviewer for this feedback on interpretive depth. This concern overlaps with Reviewer 3's comments on descriptive analysis, which we have addressed through revisions to Results sections ***pages 15-25*** (see response to Reviewer 3 above).<br><br>Specifically, the revised manuscript now provides deeper framing of:<br><br>- Tensions between NGOs/governmental actors (Outer Setting: inter-sectoral trust gaps ***page 16, lines 303-311***)<br>- Provider competition (Implementation Outcomes: adoptability barriers ***page 20, lines 406-411***)
Third, methodological transparency could be improved. The manuscript would benefit from clarification regarding coding procedures (e.g., whether double coding occurred), assessment of saturation, and reflexivity, particularly given that some interviews were conducted within the implementation team. The absence of reported barriers in the Individuals domain likely reflects social desirability bias and warrants more explicit reflection.	We thank Reviewer for highlighting these important methodological concerns, which overlap with comments from other reviewers.<br><br>These aspects have been addressed in Methods through clarification of our thematic analysis approach (please see our response to Reviewer 3 above).<br><br>***Page 10, Lines 234-244***<br><br>A reflexivity statement has been added acknowledging researchers' positionality and potential interpretive influences.

	Page 11. Lines 245-255<br><br>Regarding assessment of saturation, these are now addressed in our sampling justification.<br><br>***Page 8, Lines 178-183***
Finally, some claims of novelty should be moderated, as implementation research has been conducted in other conflict settings, even if not identical to Ukraine's context.	We thank the reviewer for this important clarification. We have moderated novelty claims in the Discussion (***page 25, lines 482-485***) to acknowledge existing implementation research in other conflict settings:<br><br>"This qualitative study expands implementation research from other conflict settings to Ukraine's active wartime context, examining factors influencing the scalability of a psychological intervention with a focus on barriers, facilitators and implementation strategies for future scale up".
Overall, the study is relevant and potentially impactful. With clearer conceptual framing, deeper analytical engagement, and strengthened methodological reporting, it would make a valuable contribution to the literature on MHPSS scalability in humanitarian settings.	We thank the reviewer for recognizing the relevance and potential impact of our study. We have carefully considered all recommendations to strengthen the manuscript. We hope these revisions now meet the expectations for contributing to MHPSS scalability literature in humanitarian settings
