# Supplementary material for: Stakeholder perspectives on the scalability of a psychological intervention for alcohol misuse and psychological distress in wartime: A qualitative study in Ukraine
Source: PLOS Ment Health. 2026 Jul 7;3(7):e0000639. doi: 10.1371/journal.pmen.0000639 (PMC13340804; doi:10.1371/journal.pmen.0000639)
Supplement: Attachment — Submitted filename: Response_to_Reviewers_auresp_2.pdf [file pmen.0000639.s008.pdf]

Response to Reviewers

Manuscript ID: PMEN-D-25-00606R1

Stakeholder perspectives on the scalability of a psychological intervention for alcohol misuse and psychological distress in wartime: A qualitative study in Ukraine

PLOS Mental Health

Dear Reviewers,

We would like to thank you for the thoughtful and constructive feedback on our manuscript. We greatly appreciate the time and effort dedicated to providing detailed suggestions, which have helped us substantially improve the clarity and analytical depth of the paper.

Below, we provide a detailed, point-by-point response to each comment raised by the reviewers. All corresponding changes have been made in the revised manuscript and are highlighted using track changes. For each revision, we have also indicated the relevant page numbers where modifications appear.

We hope that the revised version now meets the expectations of the editor and reviewers and will be considered suitable for publication in PLOS Mental Health.

Thank you again for your time and consideration.

With kind regards,

Khatia Antia

on behalf of all co-authors

**Journal Requirements:**	**Authors' response**
If the reviewer comments include a recommendation to cite specific previously published works, please review and evaluate these publications to determine whether they are relevant and should be cited. There is no requirement to cite these works unless the editor has indicated otherwise.	Thank you for this clarification. The reviewers did not recommend specific sources to cite.
Please review your reference list to ensure that it is complete and correct. If you have cited papers that have been retracted, please include the rationale for doing so in the manuscript text, or remove these references and replace them with relevant current references. Any changes to the reference list should be mentioned in the rebuttal letter that accompanies your revised manuscript. If you need to cite a retracted article, indicate the article's retracted status in the References list and also include a citation and full reference for the retraction notice.	Thank you for this important guidance. We have carefully reviewed the reference list. No retracted papers were identified. The reference list is complete and correct.

**Reviewer comment**	**Authors' response**
**Reviewer #3**	
Key issues that still need improvement (manageable revisions)<br>These are not fatal flaws, but refinements needed to improve clarity, theoretical sharpness, and publication strength:	We thank the reviewer for their thoughtful and encouraging feedback. We have carefully addressed each of the points raised below.
**1. Overly dense abstract and results presentation**	
The abstract and parts of results are overloaded with CFIR categories and long lists of barriers.<br><br>**Issue:** Reduces clarity and hides the main message<br><br>**Fix:** Focus on 3–4 core insights only, not full taxonomy listing	We thank the reviewer for this helpful observation. We have revised the abstract and the results sections into a lighter and more focused format.<br><br>We would also like to clarify that we did not aim to include the full CFIR taxonomy, including all domains and constructs. The domains currently presented were selected to reflect the needs of this study setting and will remain in the manuscript, as reducing them would result in loss of important contextual information. Retaining them is important for informing future implementation work and a planned Theory of Change workshop. However, we have reviewed the text and condensed domains with

	less influence, resulting in a more focused synthesis. Specifically, we merged less frequently mentioned barriers into broader phrases, removed repetitive findings across sections, and condensed narrative text where detailed information is already available in the tables. All findings are retained either in the main text or supplementary materials.<br><br>*(Please refer to the revised Abstract, pages 2-3, and Results section, pages 12–28)*
**2. Weak synthesis in Introduction**	
The introduction is rich but reads like a contextual review rather than a problem-driven argument.<br><br>**Issue:** Research gap is not sharply defined early enough<br>**Fix:** Clearly state:<br>what is NOT known about scalability in war contexts<br>why existing implementation evidence is insufficient	We thank the reviewer for this comment. We have revised the introduction to strengthen its problem-driven argument.<br><br>Specifically, we have sharpened the gap statement to highlight what is not known about scalability in active war contexts, distinguishing this from the existing evidence base on post-conflict and refugee settings. We have also added a paragraph that outlines the war-specific scalability determinants that remain empirically underexplored, providing a clearer rationale for the study.<br><br>Additionally, we have noted in the aims paragraph that findings will directly inform a subsequent Theory of Change workshop, in which stakeholders will collectively develop a structured scale-up pathway for CHANGE.<br><br>*(Please refer to the revised Introduction, pages 4–6)*
**3. Limited conceptual integration (CFIR + ERIC)**	
Both frameworks are used well, but:<br><br>**Issue:** They are described more than integrated analytically<br><br>**Fix:** Strengthen explanation of:<br>how CFIR → identifies determinants<br>how ERIC → translates them into strategies<br>how both jointly inform scalability conclusions	We thank the reviewer for this observation. We have strengthened the conceptual integration of CFIR and ERIC in the introduction by explicitly articulating the sequential analytical logic connecting the two frameworks. We have also noted that this integrated approach is particularly valuable in active war contexts, where implementation determinants are highly dynamic.<br><br>*(Please refer to the revised Introduction, pages 5-6)*
**4. Some sections are descriptive rather than analytical**	
Especially:<br><br>inner setting<br>policy context<br>NGO roles	We agree with the reviewer on this point and have carefully addressed it at two levels.<br><br>In the results section, we have made analytical revisions across multiple sections. In the outer setting, we merged the inter-sectoral trust gaps

**Issue:** Limited interpretation of why these factors matter for scalability theory<br>**Fix:** Add more analytical sentences linking findings to:<br><br>system capacity<br>sustainability<br>health system integration	subsection into the policy exclusion paragraph. This revision explicitly connects policy context and NGO roles to their implications for system-level scale-up. In the inner setting, we have revised the section to include analytical framing that connects workforce strain and organizational facilitators to their implications for sustained programme delivery.<br><br>In the discussion, we have added analytical interpretation of the NGO-government tension arguing that building cross-sectoral trust and establishing clear quality standards for NGO-delivered services are prerequisites for sustainable scale-up. We have also strengthened the discussion of inner setting findings by framing workforce development and wellbeing as a structural prerequisite for scalability in active war contexts.<br><br>*(Please refer to the revised Outer Setting and Inner Setting sections in Results, pages 15-20, and Discussion, pages 30-33)*
**5. "No barriers in individuals' domain" feels under-explained**	
This is unusual in implementation studies.<br><br>Issue: May suggest under-coding or over-positivity bias<br>Fix: Either:<br><br>justify strongly why no barriers emerged, OR reconsider and check whether subtle barriers were missed	We thank the reviewer for raising this important point.<br><br>We have added a transparency note at the opening of the Individuals domain section acknowledging two methodological considerations that may explain the absence of barriers.<br><br>*(Please refer to the revised individuals domain section in Results, page 20)*
**6. Results are slightly repetitive**	
Some findings repeat across:<br><br>outer setting<br>implementation outcomes<br>war-related barriers section<br>**Issue:** Reduces narrative efficiency<br><br>**Fix:** Merge overlapping themes and sharpen distinctions between sections	We thank the reviewer for this observation. We have revised the results section to reduce repetition across domains.<br><br>Specifically, we merged the inter-sectoral trust gaps subsection into the policy exclusion paragraph in the outer setting, as these findings are conceptually linked.<br><br>In the inner setting, payment instability and psychological distress findings were merged into a single analytical paragraph.<br><br>We removed the implementation outcomes section, as its content largely overlapped with other CFIR domains. Findings related to adoptability, implementability, and sustainability were

	redistributed across the respective CFIR domains according to their best thematic fit.<br><br>The war barriers narrative was condensed to a single introductory paragraph, with detailed descriptions retained in Table 2.<br><br>*(Please refer to the revised Results section, pages 12–28)*
**7. Limited theoretical takeaway in conclusion**	
**Issue:** Does not fully elevate findings to theory or policy level<br>**Fix:** Add:<br><br>1 clear theoretical contribution (e.g., scalability under war conditions expands CFIR use)<br>1 policy implication (e.g., NGO-government integration model)<br>1 scalability insight (e.g., online adaptation as core enabler)	We thank the reviewer for this suggestion.<br><br>We have substantially revised the conclusion to elevate findings to theoretical and policy levels. Specifically, we have added:<br><br>(1) a theoretical contribution noting that applying CFIR in active war contexts reveals a distinct layer of scalability determinants extending beyond those documented in stable or post-crisis settings, suggesting that existing implementation frameworks require contextual adaptation in conflict settings;<br><br>(2) a policy implication arguing that sustainable scale-up requires cross-sectoral dialogue around accreditation, licensing and long-term financing, alongside integration into national clinical standards; and<br><br>(3) a scalability insight identifying online delivery adaptation and workforce wellbeing investment as core enablers of implementation resilience.<br><br>We have also noted that findings will directly inform a subsequent Theory of Change workshop aimed at developing a structured scale-up pathway for CHANGE.<br><br>*(Please refer to the revised Conclusion, pages 35-37)*
**Reviewer #4:**	
Thank you for this revision! Overall, the manuscript is methodologically sound, theoretically grounded, and highly relevant, though several areas would benefit from further clarification and tightening.	We thank the reviewer for this encouraging assessment and for the constructive feedback provided throughout the review process. We have carefully addressed all points raised, as detailed in our responses below.
Some findings (e.g., referral pathways, stigma, financing) read more as implementation challenges than scalability-specific determinants.	We thank the reviewer for this important conceptual point. We have addressed this in introduction and discussion:

Clarifying how scalability is analytically differentiated from implementation, particularly in relation to system-level conditions (e.g., policy integration, financing, workforce capacity), would strengthen conceptual clarity.	In the introduction, we have added a clarifying phrase distinguishing implementation from scaling up.<br><br>In the discussion strengths section, we have added an analytical paragraph explicitly addressing this distinction, explaining how findings such as stigma, referral pathways, and financing constraints, while commonly encountered in implementation research, are examined here specifically for their system-level scalability implications.<br><br>*(Please refer to the revised Introduction page 4, and Discussion page 35)*
Further, the barriers of the individual domain are less developed.<br><br>More discussion could help with transparency especially if using CFIR.	We appreciate this constructive observation, which aligns with a point also raised by Reviewer #3.<br><br>We have revised the individuals domain section to improve transparency around the absence of identified barriers. We have added a methodological transparency note acknowledging two factors that may explain this finding.<br><br>We have also reflected this in the limitations section of the discussion, explicitly noting that individual-level barriers may have been mitigated by the trial context and therefore may not represent the full range of barriers likely to emerge at scale.<br><br>*(Please refer to the revised Individuals domain section, page 20, and Discussion, page 34)*
The results section is still mostly descriptive.	We thank the reviewer for this observation, which was also raised by Reviewer #3 and has been addressed accordingly.<br><br>*(Please refer to the revised Results section, pages 12–28)*
Overall, this is a strong and important manuscript. With minor revisions to enhance conceptual clarity and analytical depth, it will make a meaningful contribution to implementation science in humanitarian settings.	We thank the reviewer for this encouraging assessment and for the detailed and constructive feedback provided throughout the review process. We hope that the revisions described above adequately address the points raised and that the manuscript now meets the standard for publication in PLOS Mental Health.
