# Supplementary material for: Stakeholder perspectives on the scalability of a psychological intervention for alcohol misuse and psychological distress in wartime: A qualitative study in Ukraine
Source: PLOS Ment Health. 2026 Jul 7;3(7):e0000639. doi: 10.1371/journal.pmen.0000639 (PMC13340804; doi:10.1371/journal.pmen.0000639)
Supplement: Peer review history

# Peer review: Decision Letter 0

**DOI:** 10.1371/journal.pmen.0000639.r001

**Authors:** Shishir Paudel

4 Mar 2026

PMEN-D-25-00606

Scalability of a psychological intervention for alcohol misuse and psychological distress in wartime: A qualitative study in Ukraine

PLOS Mental Health

Dear Dr. Antia,

Thank you for submitting your manuscript to PLOS Mental Health. After careful consideration, we feel that it has merit but does not fully meet PLOS Mental Health’s publication criteria as it currently stands. Therefore, we invite you to submit a revised version of the manuscript that addresses the points raised during the review process.

Please submit your revised manuscript by 20 March 2026. If you will need more time than this to complete your revisions, please reply to this message or contact the journal office at mentalhealth@plos.org. When you’re ready to submit your revision, log on to https://www.editorialmanager.com/pmen/ and select the 'Submissions Needing Revision' folder to locate your manuscript file.

Please include the following items when submitting your revised manuscript:

- A letter that responds to each point raised by the editor and reviewer(s). You should upload this letter as a separate file labeled 'Response to Reviewers'.
- A marked-up copy of your manuscript that highlights changes made to the original version. You should upload this as a separate file labeled 'Revised Manuscript with Track Changes'.
- An unmarked version of your revised paper without tracked changes. You should upload this as a separate file labeled 'Manuscript'.

Guidelines for resubmitting your figure files are available below the reviewer comments at the end of this letter.

We look forward to receiving your revised manuscript.

Kind regards,

Shishir Paudel, MPH

Academic Editor

PLOS Mental Health

Journal Requirements:

1. Please ensure that your Ethics Statement is available in its entirety at the beginning of your Methods section, under a subheading 'Ethics Statement'.

2. We note that you have indicated that there are restrictions to data sharing for this study. For studies involving human research participant data or other sensitive data, we encourage authors to share de-identified or anonymized data. However, when data cannot be publicly shared for ethical reasons, we allow authors to make their data sets available upon request. For information on unacceptable data access restrictions, please see http://journals.plos.org/plosone/s/data-availability#loc-unacceptable-data-access-restrictions.

Before we proceed with your manuscript, please address the following prompts:

a) If there are ethical or legal restrictions on sharing a de-identified data set, please explain them in detail (e.g., data contain potentially identifying or sensitive patient information, data are owned by a third-party organization, etc.) and who has imposed them (e.g., a Research Ethics Committee or Institutional Review Board, etc.). Please also provide contact information for a data access committee, ethics committee, or other institutional body to which data requests may be sent.

b) If there are no restrictions, please upload the minimal anonymized data set necessary to replicate your study findings to a stable, public repository and provide us with the relevant URLs, DOIs, or accession numbers. Please see http://www.bmj.com/content/340/bmj.c181.long for guidelines on how to de-identify and prepare clinical data for publication. For a list of recommended repositories, please see https://journals.plos.org/plosone/s/recommended-repositories. You also have the option of uploading the data as Supporting Information files, but we would recommend depositing data directly to a data repository if possible.

Please update your Data Availability statement in the submission form accordingly.

If the reviewer comments include a recommendation to cite specific previously published works, please review and evaluate these publications to determine whether they are relevant and should be cited. There is no requirement to cite these works unless the editor has indicated otherwise.

Additional Editor Comments (if provided):

Please focus on methodological transparency as suggested by the Reviewers.

[Note: HTML markup is below. Please do not edit.]

Reviewers' comments:

Reviewer's Responses to Questions

**Comments to the Author**

1. Does this manuscript meet PLOS Mental Health’s publication criteria? Is the manuscript technically sound, and do the data support the conclusions? The manuscript must describe methodologically and ethically rigorous research with conclusions that are appropriately drawn based on the data presented.

Reviewer #1: Yes

Reviewer #2: Yes

Reviewer #3: Yes

Reviewer #4: Partly

**********

2. Has the statistical analysis been performed appropriately and rigorously?

Reviewer #1: N/A

Reviewer #2: Yes

Reviewer #3: Yes

Reviewer #4: N/A

**********

3. Have the authors made all data underlying the findings in their manuscript fully available (please refer to the Data Availability Statement at the start of the manuscript PDF file)?

The PLOS Data policy requires authors to make all data underlying the findings described in their manuscript fully available without restriction, with rare exception. The data should be provided as part of the manuscript or its supporting information, or deposited to a public repository. For example, in addition to summary statistics, the data points behind means, medians and variance measures should be available. If there are restrictions on publicly sharing data—e.g. participant privacy or use of data from a third party—those must be specified.

Reviewer #1: Yes

Reviewer #2: Yes

Reviewer #3: Yes

Reviewer #4: Yes

**********

4. Is the manuscript presented in an intelligible fashion and written in standard English?

PLOS Mental Health does not copyedit accepted manuscripts, so the language in submitted articles must be clear, correct, and unambiguous. Any typographical or grammatical errors should be corrected at revision, so please note any specific errors here.

Reviewer #1: Yes

Reviewer #2: Yes

Reviewer #3: Yes

Reviewer #4: Yes

**********

5. Review Comments to the Author

Please use the space provided to explain your answers to the questions above. You may also include additional comments for the author, including concerns about dual publication, research ethics, or publication ethics. (Please upload your review as an attachment if it exceeds 20,000 characters)

Reviewer #1: Thank you to the authors and the PLOS editorial team for giving me the opportunity to review this vital and timely manuscript; I believe it contributes significantly to the field of war and emergency mental health interventions, particularly given the ongoing situation in Ukraine.

Firstly, I'd like to commend the decision to adapt a robustly tried and tested MH intervention, the WHO's problem management plus programme and mhGAP, for alcohol misuse in an active war zone. Second, the overall qualitative findings lend well to wider implementation of the CHANGE intervention as the study interviews a broad range of stakeholders involved in the delivery, implementation, and reception of the intervention.

I have highlighted a few suggestions and edits below:

1. Introduction section:

- Lines 59 - 60; you can also consider drawing on other conflict-affected populations here that might be more relevant to war activities; for example, the American opioid crisis and its particular effects on the American veteran population.

- Line 78 - 79; would it be possible to give the measurements for alcohol use in units? Units are widely used for reporting alcohol consumption in the UK, US and so on, which is where I assume a majority of PLOS MH's readership is located.

- Line 80; as this study sought to adapt and deliver this intervention to men, it is advisable in this section of the introduction to provide a bit more context as to why alcohol consumption as a coping strategy is more widespread/prevalent in men compared to women. I believe this is an important premise to provide in order to justify the subsequent sampling decisions.

- Line 120 - 128; I agree with the decision to use the CFIR framework; I believe it is well suited to determining the implementation barriers and facilitators of CHANGE in the context of war in Ukraine. However, I am wondering if it would be possible to give an example of a previous study where the CFIR was used in war or similar contexts? this would help with contextualising the on-the ground reality of conducting a CFIR evaluation.

- Line 134-143; this paragraph includes information about the intervention's methodology and delivery and therefore would be better placed into the study's materials and methods section.

2. Materials and methods;

- Line 155; I would recommend splitting up this section into two; one section being the study design, and second being the sample. For the sample section, it might be helpful to include information about who (from which org, in what capacity) was interviewed, and for the study design section to provide information on why (for example, 'adopters' were needed to understand the extent and features of integrating CHANGE into preexisting practices). Nevertheless, this can be considered a stylistic choice, so feel free to amend or disregard as you see fit.

- Line 209; was the same certified translator used for all the interviews that needed translating? this is useful information to provide as using a variety of translators from the same service might result in differences in how certain text or non text cues might be interpreted. Perhaps something to consider highlighting in the discussion section as well.

- Line 217; would it be possible to provide a bit more information about how the research team collaborated in thematically analysing the findings? for example, if any researcher reflexivity practices were followed, if there was a first and second coder, how collective decisions were made regarding top level codes, validity checks, etc. This information, if made available, can greatly boost the perceived validity of your findings.

3. Results and Discussion section:

- Line 235; I assume this means a majority of the interviewees apart from the stakeholders were female? As the CHANGE programme is designed to specifically address excessive alcohol consumption in men, it would be helpful for the study's discussion section to identify this as a limitation as one could argue certain lived experience aspects of being a male might be useful to understanding the barriers and facilitators of implementing CHANGE. i.e, when it comes to assumptions about uptake, how to address stigma, etc.

- Table 1; Construct names 'Culture' and 'Available resources'; how is psychological distress amongst the CHANGE implementation team handled? Would be helpful for the methods section to include some detail about the supervision and/or pastoral support provided to implementers of CHANGE.

- Line 421 to 423 and line 627 to 628; is it possible to provide some contextual information about what other intervention offerings are available? For example, include in the introduction section which organisations are offering similar or comparable psychosocial interventions and the characteristics of these interventions. That would be helpful to understanding the premise of this section.

- Line 459 to 464; as the recipients of CHANGE were identified in the introduction section to be largely internally displaced populations, I am wondering if it might be useful to provide information in the discussion section on how the CHANGE programme can be adapted further to account for the frequent disruptions to sessions that recipients might encounter. A few examples could be; increased flexibility on the part of treatment providers in the sessions, making the sessions independent and not consecutive so there is no knock-on impact from missing a session or two, flexible delivery formats, more emphasis on homework tasks so longitudinal progress of the recipient is retained, etc.

- Line 590; stigma from accessing family or community-based MH interventions has been identified as a significant barrier by stakeholders in this study; I imagine a proportion of this stigma also arises from the prevalent gender roles and expectations for how men are 'expected' to cope with adversity compared to women. nevertheless, as the majority of the stakeholders interviewed in this study identified as female, it would be helpful to add a recommendation here for future research into CHANGE to be conducted with male stakeholders to understand if/how gender can impact uptake/effectiveness/acceptability of the intervention.

Reviewer #2: This is an important and timely piece of research that explores the role of AUD in a specific traumatised population. I would like to see a little more detail in respect of “scaling up”. The premise of the study is to look at how tenable scaling up to war settings (lines 144 onwards) however I feel it needs a little more exploration in the introduction. Maybe even a little more discussion of ecological validity of such treatment programs.

The data is as expected and its important to look at these problems in the context of a real world application.

I would like to note that I enjoyed reviewing this manuscript.

Introduction

The introduction outlines the problem in its wider context and speaks to the issues in delivering a successful intervention-based treatment program. I might suggest that an overview paragraph is included at the beginning of this section before the authors begin to outline the issues to be addressed.

Methods

This section describes a classic thematic analysis approach. Attention to ethical detail especially in respect to enhanced approach to consent to use quotes is commendable.

While it seems that every attempt has been made to reduce subjective bias in the coding of the data would it have been helpful to guide research team discussions around the themes based on multiple coding from the team?

Results

The data is compelling and clearly identifies a disconnect withing the context of the scalability of the program to be effective. The tables provide a nice overview of the data. As with all thematic analysis it is often hard to disentangle the interpretive nature of the data. However there is a well-defined framework with the results section. This could have been made a little more explicit to strengthen the data presented.

Discussion

One suggestion that is probably one of those reviewers’ comments that are aligned with “but we didn’t study that”, might be useful for future work is to look at this within the theoretical framework of Activity Theory. I think it might give a nice framework to try and look at how different stakeholders could be aligned.

Reviewer #3: This manuscript addresses a highly relevant and timely topic, namely the scalability of a psychological intervention for alcohol misuse and psychological distress in the context of active war in Ukraine. The study has clear potential to contribute to the global mental health and implementation science literature, particularly for humanitarian and conflict-affected settings. However, while the topic and overall approach are appropriate for PLOS Mental Health, several methodological, analytical, and reporting issues currently limit the strength of the evidence and the validity of the conclusions. Substantial revision is required to improve clarity, rigor, and alignment between the data presented and the claims made.

With respect to the title and abstract (pp. 1–2), the manuscript frames the study as an assessment of “scalability,” yet the data primarily capture stakeholder perceptions and expectations rather than empirical evidence of scale-up feasibility or effectiveness. This creates a degree of conceptual overstatement, particularly in the abstract, where conclusions imply readiness for scale-up. The authors are encouraged to refine the title and abstract to more clearly reflect the exploratory and perception-based nature of the findings, and to explicitly distinguish between perceived facilitators and barriers and demonstrated scalability outcomes.

The introduction (pp. 3–5) provides a comprehensive overview of alcohol misuse, psychological distress, and the Ukrainian wartime context, but it is at times overly detailed and repetitive. While the epidemiological background is strong, the specific knowledge gap addressed by this study could be articulated more sharply. The authors would strengthen this section by streamlining background content, reducing repetition, and more clearly positioning the study within existing literature on implementation and scale-up of mental health and psychosocial support interventions in humanitarian settings. Explicit justification for the use of qualitative methods to address the stated research questions would also improve conceptual coherence.

The use of the Consolidated Framework for Implementation Research and the ERIC implementation strategies framework (pp. 5–6) is appropriate; however, the rationale for selecting these frameworks is presented descriptively rather than critically. The manuscript would benefit from a clearer explanation of why CFIR and ERIC are particularly suitable for examining scalability in wartime or humanitarian contexts, and how they compare to alternative implementation frameworks. Greater clarity on how the study’s research questions map onto specific CFIR domains would also enhance transparency and conceptual alignment.

In the Methods section describing study design and sampling (pp. 6–7), the qualitative approach is appropriate, but key methodological details are insufficiently reported. The manuscript does not clearly justify the sample size, nor does it explain how thematic saturation was assessed or whether saturation was a guiding principle. Additionally, the implications of recruiting participants primarily from implementing and policy-related organizations are not fully addressed. The authors should clarify how participant roles and institutional affiliations may have shaped responses, and more explicitly acknowledge any limitations this introduces for generalizability and interpretation.

Regarding data collection procedures (pp. 7–8), the authors provide a basic description of interview logistics, consent, and translation processes. However, there is limited reflection on the challenges of conducting qualitative research during active conflict, including how war-related stress, insecurity, or power dynamics may have influenced participation and responses. The authors are encouraged to include a brief reflective discussion on these issues and to further clarify how translation accuracy and meaning equivalence were ensured during transcription and analysis.

The data analysis section (pp. 8–9) lacks sufficient detail to fully assess analytic rigor. While the use of thematic analysis guided by CFIR is stated, the manuscript does not clearly specify whether a reflexive or code-reliability approach was employed, nor how analytic decisions were documented and validated. To strengthen trustworthiness, the authors should elaborate on steps taken to enhance rigor, such as team-based interpretation, audit trails, or reflexive discussion. A brief reflexivity statement acknowledging the researchers’ positionality and potential interpretive influences would further strengthen the analysis.

In the Results section (pp. 9–13), the findings are generally well organized according to CFIR domains, but several themes overlap conceptually, leading to redundancy. In addition, some sections remain largely descriptive, with limited interpretive depth. While participant quotations are valuable, they occasionally dominate without sufficient analytical synthesis. The authors are encouraged to consolidate overlapping themes, strengthen interpretive commentary explaining why identified barriers and facilitators matter for scalability, and ensure that quotations consistently support clear analytic points rather than standing alone as illustrative material.

The Discussion section (pp. 13–16) appropriately situates the findings within the broader literature, but at times overextends conclusions beyond what the qualitative data can support. In particular, claims regarding scalability and policy relevance should be more cautiously framed, given that the study captures perceptions rather than tested scale-up outcomes. The discussion would benefit from clearer differentiation between implications for future research, considerations for implementation planning, and broader policy relevance. Stronger engagement with international literature on task-sharing and humanitarian mental health would also enhance the contribution.

Ethical considerations and data availability (pp. 8, 16) are addressed, but somewhat superficially given the sensitivity of conducting research in a wartime context. The manuscript would be strengthened by deeper reflection on ethical challenges such as participant vulnerability, informed consent under duress, and researcher responsibility. In addition, while restrictions on data sharing are understandable, the Data Availability Statement should more clearly specify what de-identified materials (e.g., codebooks or analytic summaries) could be made available upon reasonable request, in line with PLOS data transparency expectations.

Finally, the conclusion (p. 16) largely reiterates the study’s findings but does not sufficiently temper claims regarding scalability. The authors are encouraged to reframe the conclusion to emphasize the study’s exploratory contribution to understanding perceived barriers and facilitators, rather than implying readiness for immediate large-scale implementation. Clearly positioning the findings as hypothesis-generating would better align conclusions with the qualitative evidence presented.

In summary, this manuscript addresses an important and under-researched topic with significant potential relevance for global mental health practice in conflict settings. However, major revisions are required to improve methodological transparency, analytical rigor, and alignment between findings and conclusions. Addressing the issues outlined above would substantially strengthen the manuscript and its suitability for publication in PLOS Mental Health.

Reviewer #4: This manuscript addresses a timely and important topic: the scalability of a psychological intervention for alcohol misuse and psychological distress during active wartime in Ukraine. The use of CFIR and ERIC frameworks, along with perspectives from implementers, adopters, and maintainers, is a clear strength. The paper offers valuable contextual insights into how war-related disruptions, stigma, funding instability, and NGO–government dynamics shape implementation.

However, several areas require strengthening before the manuscript is suitable for publication.

First, the concept of “scalability” needs clearer differentiation from general implementation, feasibility, or sustainability. While barriers and facilitators are well described, it is not always clear how these specifically relate to scale-up beyond routine delivery. Clarifying what scale would concretely mean in this context (e.g., national integration, public financing, primary care embedding) would strengthen the contribution.

Second, the analysis is largely descriptive and closely follows CFIR domains. Greater interpretive depth would improve the manuscript. For example, tensions between NGOs and governmental actors, concerns about professional legitimacy, competition among providers, and stigma in the context of masculinity and mobilization fears could be more critically examined. Moving beyond categorization toward theoretical insight would elevate the paper.

Third, methodological transparency could be improved. The manuscript would benefit from clarification regarding coding procedures (e.g., whether double coding occurred), assessment of saturation, and reflexivity, particularly given that some interviews were conducted within the implementation team. The absence of reported barriers in the Individuals domain likely reflects social desirability bias and warrants more explicit reflection.

Finally, some claims of novelty should be moderated, as implementation research has been conducted in other conflict settings, even if not identical to Ukraine’s context.

Overall, the study is relevant and potentially impactful. With clearer conceptual framing, deeper analytical engagement, and strengthened methodological reporting, it would make a valuable contribution to the literature on MHPSS scalability in humanitarian settings.

**********

6. PLOS authors have the option to publish the peer review history of their article (what does this mean?). If published, this will include your full peer review and any attached files.

**Do you want your identity to be public for this peer review?**  If you choose “no”, your identity will remain anonymous but your review may still be made public.

For information about this choice, including consent withdrawal, please see our Privacy Policy.

Reviewer #1: **Yes:**  Sasha Menon

Reviewer #2: No

Reviewer #3: **Yes:**  Jamal Jumanne Athuman

Reviewer #4: No

**********

[NOTE: If reviewer comments were submitted as an attachment file, they will be attached to this email and accessible via the submission site. Please log into your account, locate the manuscript record, and check for the action link "View Attachments". If this link does not appear, there are no attachment files.]

-->Figure Resubmissions:

While revising your submission, we strongly recommend that you use PLOS’s NAAS tool (https://ngplosjournals.pagemajik.ai/artanalysis) to test your figure files. NAAS can convert your figure files to the TIFF file type and meet basic requirements (such as print size, resolution), or provide you with a report on issues that do not meet our requirements and that NAAS cannot fix.

After uploading your figures to PLOS’s NAAS tool - https://ngplosjournals.pagemajik.ai/artanalysis, NAAS will process the files provided and display the results in the "Uploaded Files" section of the page as the processing is complete. If the uploaded figures meet our requirements (or NAAS is able to fix the files to meet our requirements), the figure will be marked as "fixed" above. If NAAS is unable to fix the files, a red "failed" label will appear above. When NAAS has confirmed that the figure files meet our requirements, please download the file via the download option, and include these NAAS processed figure files when submitting your revised manuscript.

# Author comment: Author response to Decision Letter 1

**DOI:** 10.1371/journal.pmen.0000639.r002

31 Mar 2026

Attachment Submitted filename: Response to Reviewers.pdf

# Peer review: Decision Letter 1

**DOI:** 10.1371/journal.pmen.0000639.r003

**Authors:** Shishir Paudel, Shishir Paudel

7 May 2026

PMEN-D-25-00606R1

Stakeholder perspectives on the scalability of a psychological intervention for alcohol misuse and psychological distress in wartime: A qualitative study in Ukraine

PLOS Mental Health

Dear Dr. Antia,

Thank you for submitting your manuscript to PLOS Mental Health. After careful consideration, we feel that it has merit but does not fully meet PLOS Mental Health’s publication criteria as it currently stands. Therefore, we invite you to submit a revised version of the manuscript that addresses the points raised during the review process.

Please ensure that your decision is justified on PLOS Mental Health’s publication criteria and not, for example, on novelty or perceived impact.

Please submit your revised manuscript by Jun 06 2026 11:59PM. If you will need more time than this to complete your revisions, please reply to this message or contact the journal office at mentalhealth@plos.org. When you're ready to submit your revision, log on to https://www.editorialmanager.com/pmen/ and select the 'Submissions Needing Revision' folder to locate your manuscript file.

Please include the following items when submitting your revised manuscript:

- A letter that responds to each point raised by the editor and reviewer(s). You should upload this letter as a separate file labeled 'Response to Reviewers'.
- A marked-up copy of your manuscript that highlights changes made to the original version. You should upload this as a separate file labeled 'Revised Manuscript with Track Changes'.
- An unmarked version of your revised paper without tracked changes. You should upload this as a separate file labeled 'Manuscript'.

Guidelines for resubmitting your figure files are available below the reviewer comments at the end of this letter.

As the corresponding author, your ORCID iD is verified in the submission system and will appear in the published article. PLOS supports the use of ORCID, and we encourage all coauthors to register for an ORCID iD and use it as well. Please encourage your coauthors to verify their ORCID iD within the submission system before final acceptance, as unverified ORCID iDs will not appear in the published article. *Only*  the individual author can complete the verification step; PLOS staff *cannot*  verify ORCID iDs on behalf of authors.

We look forward to receiving your revised manuscript.

Kind regards,

Shishir Paudel, MPH

Academic Editor

PLOS Mental Health

Journal Requirements:

If the reviewer comments include a recommendation to cite specific previously published works, please review and evaluate these publications to determine whether they are relevant and should be cited. There is no requirement to cite these works unless the editor has indicated otherwise.

Please review your reference list to ensure that it is complete and correct. If you have cited papers that have been retracted, please include the rationale for doing so in the manuscript text, or remove these references and replace them with relevant current references. Any changes to the reference list should be mentioned in the rebuttal letter that accompanies your revised manuscript. If you need to cite a retracted article, indicate the article’s retracted status in the References list and also include a citation and full reference for the retraction notice.

Additional Editor Comments (if provided):

[Note: HTML markup is below. Please do not edit.]

Reviewers' comments:

Reviewer's Responses to Questions

**Comments to the Author**

1. If the authors have adequately addressed your comments raised in a previous round of review and you feel that this manuscript is now acceptable for publication, you may indicate that here to bypass the “Comments to the Author” section, enter your conflict of interest statement in the “Confidential to Editor” section, and submit your "Accept" recommendation.

Reviewer #3: All comments have been addressed

Reviewer #4: (No Response)

**********

2. Does this manuscript meet PLOS Mental Health’s publication criteria? Is the manuscript technically sound, and do the data support the conclusions? The manuscript must describe methodologically and ethically rigorous research with conclusions that are appropriately drawn based on the data presented.

Reviewer #3: Yes

Reviewer #4: Yes

**********

3. Has the statistical analysis been performed appropriately and rigorously?

Reviewer #3: Yes

Reviewer #4: N/A

**********

4. Have the authors made all data underlying the findings in their manuscript fully available (please refer to the Data Availability Statement at the start of the manuscript PDF file)?

The PLOS Data policy requires authors to make all data underlying the findings described in their manuscript fully available without restriction, with rare exception. The data should be provided as part of the manuscript or its supporting information, or deposited to a public repository. For example, in addition to summary statistics, the data points behind means, medians and variance measures should be available. If there are restrictions on publicly sharing data—e.g. participant privacy or use of data from a third party—those must be specified.

Reviewer #3: Yes

Reviewer #4: Yes

**********

5. Is the manuscript presented in an intelligible fashion and written in standard English?

PLOS Mental Health does not copyedit accepted manuscripts, so the language in submitted articles must be clear, correct, and unambiguous. Any typographical or grammatical errors should be corrected at revision, so please note any specific errors here.

Reviewer #3: Yes

Reviewer #4: Yes

**********

6. Review Comments to the Author

Please use the space provided to explain your answers to the questions above. You may also include additional comments for the author, including concerns about dual publication, research ethics, or publication ethics. (Please upload your review as an attachment if it exceeds 20,000 characters)

Reviewer #3: Overall strengths of the article

1. High relevance and timeliness

The study addresses a very important and current issue: scaling psychological interventions for alcohol misuse and distress during war in Ukraine. This is a strong contribution because evidence from active conflict settings is still limited.

2. Strong conceptual framing

The use of CFIR and ERIC frameworks is appropriate and gives the study a solid implementation science structure. This strengthens its academic credibility and makes findings useful beyond Ukraine.

3. Rich qualitative dataset

The study includes diverse stakeholders (implementers, adopters, maintainers), which is a key strength for scalability research. The inclusion of policy-level actors is particularly valuable.

4. Detailed and transparent methods

The authors clearly describe:

sampling strategy

data collection process

translation procedures

reflexivity

ethical considerations

This increases trustworthiness.

5. Important real-world insights

The findings are highly relevant for humanitarian mental health programming:

war-related disruption of services

funding instability

stigma and referral barriers

importance of NGO–government partnerships

These are practically useful for scaling interventions globally.

6. Strong linkage between implementation science and field realities

The integration of CFIR domains with real stakeholder experiences is well executed, especially in the results section.

Key issues that still need improvement (manageable revisions)

These are not fatal flaws, but refinements needed to improve clarity, theoretical sharpness, and publication strength:

1. Overly dense abstract and results presentation

The abstract and parts of results are overloaded with CFIR categories and long lists of barriers.

Issue: Reduces clarity and hides the main message

Fix: Focus on 3–4 core insights only, not full taxonomy listing

2. Weak synthesis in Introduction

The introduction is rich but reads like a contextual review rather than a problem-driven argument.

Issue: Research gap is not sharply defined early enough

Fix: Clearly state:

what is NOT known about scalability in war contexts

why existing implementation evidence is insufficient

3. Limited conceptual integration (CFIR + ERIC)

Both frameworks are used well, but:

Issue: They are described more than integrated analytically

Fix: Strengthen explanation of:

how CFIR → identifies determinants

how ERIC → translates them into strategies

how both jointly inform scalability conclusions

4. Some sections are descriptive rather than analytical

Especially:

inner setting

policy context

NGO roles

Issue: Limited interpretation of why these factors matter for scalability theory

Fix: Add more analytical sentences linking findings to:

system capacity

sustainability

health system integration

5. “No barriers in individuals domain” feels under-explained

This is unusual in implementation studies.

Issue: May suggest under-coding or over-positivity bias

Fix: Either:

justify strongly why no barriers emerged, OR

reconsider and check whether subtle barriers were missed

6. Results are slightly repetitive

Some findings repeat across:

outer setting

implementation outcomes

war-related barriers section

Issue: Reduces narrative efficiency

Fix: Merge overlapping themes and sharpen distinctions between sections

7. Limited theoretical takeaway in conclusion

The conclusion is correct but generic.

Issue: Does not fully elevate findings to theory or policy level

Fix: Add:

1 clear theoretical contribution (e.g., scalability under war conditions expands CFIR use)

1 policy implication (e.g., NGO-government integration model)

1 scalability insight (e.g., online adaptation as core enabler)

Reviewer #4: Thank you for this revision! Overall, the manuscript is methodologically sound, theoretically grounded, and highly relevant, though several areas would benefit from further clarification and tightening. Some findings (e.g., referral pathways, stigma, financing) read more as implementation challenges than scalability-specific determinants. Clarifying how scalability is analytically differentiated from implementation, particularly in relation to system-level conditions (e.g., policy integration, financing, workforce capacity), would strengthen conceptual clarity. Further, the barriers of the individual domain are less developed. More discussion could help with transparency especially if using CFIR. The results section is still mostly descriptive. Overall, this is a strong and important manuscript. With minor revisions to enhance conceptual clarity and analytical depth, it will make a meaningful contribution to implementation science in humanitarian settings.

**********

7. PLOS authors have the option to publish the peer review history of their article (what does this mean?). If published, this will include your full peer review and any attached files.

**Do you want your identity to be public for this peer review?**  If you choose “no”, your identity will remain anonymous but your review may still be made public.

For information about this choice, including consent withdrawal, please see our Privacy Policy.

Reviewer #3: No

Reviewer #4: No

**********

[NOTE: If reviewer comments were submitted as an attachment file, they will be attached to this email and accessible via the submission site. Please log into your account, locate the manuscript record, and check for the action link "View Attachments". If this link does not appear, there are no attachment files.]

-->Figure Resubmissions:

While revising your submission, we strongly recommend that you use PLOS’s NAAS tool (https://ngplosjournals.pagemajik.ai/artanalysis) to test your figure files. NAAS can convert your figure files to the TIFF file type and meet basic requirements (such as print size, resolution), or provide you with a report on issues that do not meet our requirements and that NAAS cannot fix.

After uploading your figures to PLOS’s NAAS tool - https://ngplosjournals.pagemajik.ai/artanalysis, NAAS will process the files provided and display the results in the "Uploaded Files" section of the page as the processing is complete. If the uploaded figures meet our requirements (or NAAS is able to fix the files to meet our requirements), the figure will be marked as "fixed" above. If NAAS is unable to fix the files, a red "failed" label will appear above. When NAAS has confirmed that the figure files meet our requirements, please download the file via the download option, and include these NAAS processed figure files when submitting your revised manuscript.

# Author comment: Author response to Decision Letter 2

**DOI:** 10.1371/journal.pmen.0000639.r004

22 May 2026

Attachment Submitted filename: Response_to_Reviewers_auresp_2.pdf

# Editor report: Decision Letter 2

**DOI:** 10.1371/journal.pmen.0000639.r005

**Authors:** Shishir Paudel, Shishir Paudel, Shishir Paudel

29 May 2026

Stakeholder perspectives on the scalability of a psychological intervention for alcohol misuse and psychological distress in wartime: A qualitative study in Ukraine

PMEN-D-25-00606R2

Dear Dr. Fuhr,

We are pleased to inform you that your manuscript 'Stakeholder perspectives on the scalability of a psychological intervention for alcohol misuse and psychological distress in wartime: A qualitative study in Ukraine' has been provisionally accepted for publication in PLOS Mental Health.

Before your manuscript can be formally accepted you will need to complete some formatting changes, which you will receive in a follow up email. A member of our team will be in touch with a set of requests.

Please note that your manuscript will not be scheduled for publication until you have made the required changes, so a swift response is appreciated.

IMPORTANT: The editorial review process is now complete. PLOS will only permit corrections to spelling, formatting or significant scientific errors from this point onwards. Requests for major changes, or any which affect the scientific understanding of your work, will cause delays to the publication date of your manuscript.

If your institution or institutions have a press office, please notify them about your upcoming paper to help maximize its impact. If they'll be preparing press materials, please inform our press team as soon as possible -- no later than 48 hours after receiving the formal acceptance. Your manuscript will remain under strict press embargo until 2 pm Eastern Time on the date of publication. For more information, please contact mentalhealth@plos.org.

Thank you again for supporting Open Access publishing; we are looking forward to publishing your work in PLOS Mental Health.

Best regards,

Shishir Paudel,

Academic Editor

PLOS Mental Health

***********************************************************

Reviewer Comments (if any, and for reference):
